# Suppression of tumor metastasis by a *RECK*-activating small molecule

**DOI:** 10.1038/s41598-022-06288-3

**Published:** 2022-02-11

**Authors:** Yoko Yoshida, Kanako Yuki, Shingo Dan, Kanami Yamazaki, Makoto Noda

**Affiliations:** 1 Department of Molecular Oncology, Kyoto University Graduate School of Medicine, Sakyo-ku, Kyoto, 606-8501 Japan; 2 Division of Molecular Pharmacology, Cancer Chemotherapy Center, Japanese Foundation for Cancer Research, Koto-ku, Tokyo, 135-8550 Japan

**Keywords:** Cancer, Cell biology, Drug discovery

## Abstract

*RECK* encodes a membrane-anchored protease-regulator which is often downregulated in a wide variety of cancers, and reduced *RECK* expression often correlates with poorer prognoses. In mouse models, forced expression of RECK in tumor xenografts results in suppression of tumor angiogenesis, invasion, and metastasis. *RECK* mutations, however, are rare in cancer genomes, suggesting that agents that re-activate dormant *RECK* may be of clinical value. We found a potent *RECK*-inducer, DSK638, that inhibits spontaneous lung metastasis in our mouse xenograft model. Induction of *RECK* expression involves SP1 sites in its promoter and may be mediated by KLF2. DSK638 also upregulates MXI1, an endogenous MYC-antagonist, and inhibition of metastasis by DSK638 is dependent on both RECK and MXI1. This study demonstrates the utility of our approach (using a simple reporter assay followed by multiple phenotypic assays) and DSK638 itself (as a reference compound) in finding potential metastasis-suppressing drugs.

## Introduction

Invasion and metastasis make tumors uncontrollable^1–4^. Drugs that prevent these processes should be of clinical value^5^. *RECK* was initially isolated as a transformation-suppressor gene against the *v-K-RAS* oncogene and found to encode a membrane-anchored protease-regulator^6–11^. *RECK* is downregulated in a wide variety of cancers, and reduced *RECK* expression often correlates with poorer prognoses^12–14^.

In mouse xenograft models, forced expression of RECK in tumor cells results in suppression of tumor angiogenesis, invasion, and metastasis^7^, suggesting that RECK-downregulation plays a causal role in tumorigenesis. *RECK* mutations, however, are rare in cancer genomes, suggesting that RECK expression is transcriptionally and/or epigenetically suppressed and agents that can re-activate dormant *RECK* in cancer cells may be of clinical value.

In this study, we performed a high throughput screen using a *RECK*-promoter-reporter assay and found a potent *RECK*-inducer, DSK638, that inhibits metastasis in a mouse xenograft model. We determined the cis-elements responsible for DSK638-response and identified a candidate transcription factor involved in this response. Using DSK638 as a positive control, we established assay systems (quantitative reversion assay and suspension culture) to evaluate the potential anti-oncogenic/anti-metastatic activities of test chemicals in vitro. Lastly, a series of transcriptome analyses were performed to gain insights into the molecular mechanisms of metastasis suppression by DSK638, and involvement of candidate genes was experimentally validated.

## Results

### Identification of DSK638 as a RECK-inducing small molecule

**Figure 1:**
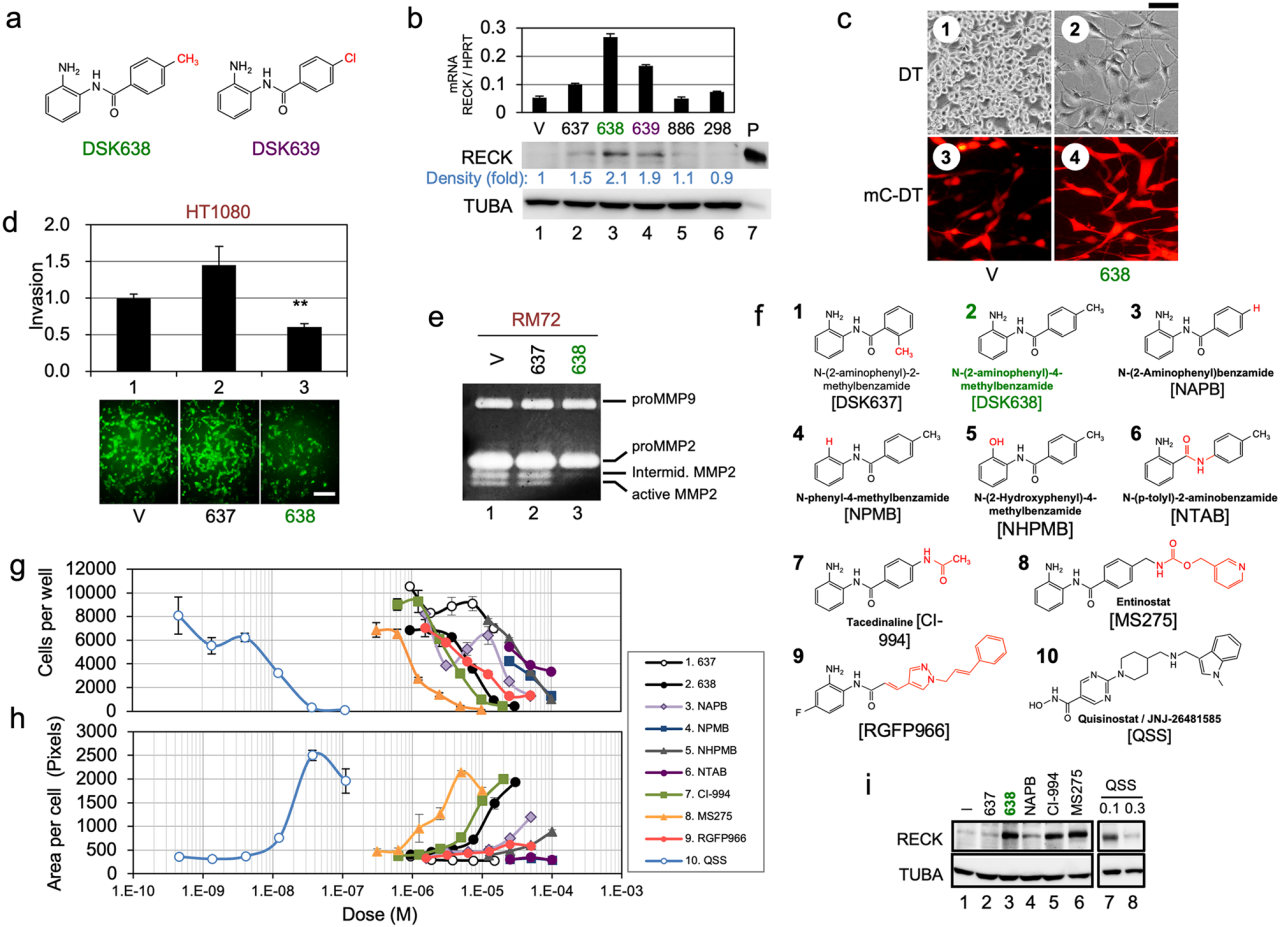
Detection and initial characterization of *RECK*-activating compounds. (**a**) Structures of two compounds exhibiting *RECK*-inducing activity. (**b**) Validation of RECK-inducing activities of five compounds selected by high throughput screening. HT1080 cells were exposed to vehicle (V) or a compound (code number) at 10 µM for 48 h. Total RNA and cell lysate were subjected to qRT-PCR (top bar graph) and immunoblot assay (bottom photographs), respectively, to detect *RECK* mRNA and RECK protein. qRT-PCR data were normalized against HPRT (internal control) and divided by the data for vehicle-treated samples (mean + s.e.m., n = 2). The number below the RECK immunoblot panel represents RECK band density normalized against $\upalpha$-tubulin (TUBA; internal control). (**c**) Flat reversion of *v-K-ras*-transformed cells by DSK638. The parental DT cells (1, 2) or DSK4b cells (mCherry-expressing DT cells) (3, 4) were incubated in medium containing vehicle (1, 3) or 10 µM DSK638 (2, 4) for 72 h and photographed under a phase-contrast (1, 2) or fluorescence (3, 4) microscope. Scale bar: 100 µm. (**d**)**.** Suppression of Matrigel invasion of HT1080 cells by DSK638. Cells pre-treated with vehicle (V), DSK637 (637), or DSK638 (638) were fluorescently labeled and subjected to Matrigel invasion assay in medium containing the same test compound. The relative number of cells that migrated across the Matrigel layer in 24 h (lower panels; scale bar: 200 µm.) were quantified by image analysis using ImageJ. The value relative to the vehicle-treated cells are presented in the top bar graph (mean + s.e.m., n = 4) **P < 0.01. The experiments were repeated twice with similar results. (**e**) Suppression of proMMP2-activation by DSK638. Gelatin zymography was performed using culture supernatants prepared from RM72 cells treated with vehicle, 10 µM DSK637, or 10 µM DSK638. Note the substantially lower intensity of the bottom two bands, which correspond to intermediate and active MMP2, respectively. (**f**) Compounds tested by qRev assay. Abbreviations or short names are shown in parentheses. (**g, h**) qRev assay. DSK4b cells were exposed to the indicated drug at various concentrations for 60 h, and the relative numbers of nuclei (g) and the relative area per cell (h) were determined: experiments were performed in triplicate. (**i**) Effects of the compounds showing the highest activity in the qRev assay on RECK protein expression in HT1080 cells. HT1080 cells were exposed to medium (-) or the medium containing the indicated compound for 48 h, and the cell lysates were subjected to immunoblot assay to detect RECK (upper panel) and $\upalpha$-tubulin (lower panel). The concentration of each drug was 10 µM except for QSS whose concentrations were 0.1 µM (lane 12) and 0.3 µM (lane 13).

A *RECK*-promoter reporter assay^15^, adapted to the human fibrosarcoma cell line HT1080, was used to screen a chemical library consisting of 65,000 small molecules (unpublished). This screen yielded two compounds (#638 and #639; Fig. 1a) that increased the level of RECK protein more than twofold (Fig. 1b). The most active compound (DSK638) exhibited bio-activities similar to RECK, such as induction of flat reversion in *v-K-RAS*-transformed cells (Fig. 1c), suppression of Matrigel-invasion by HT1080 cells (Fig. 1d), and inhibition of pro-MMP2 activation (Fig. 1e). This compound binds histone deacetylases (HDACs)^16^, and we confirmed its capability to inhibit HDAC1 and HDAC3 (IC_50_: ~ 5 µM for HDAC1, ~ 10 µM for HDAC3). We also examined the effect of DSK638 on a panel of 39 human cancer cell lines^17–19^; the spectrum of cell inhibition by DSK638 was similar to those of several HDAC inhibitors (Supplementary Table S2). We next characterized a series of compounds structurally and/or functionally related to DSK638 (Fig. 1f). Well-characterized benzamide-type HDAC inhibitors, CI-994/Tacedinaline^20^, MS275/Entinostat^21^, and JNJ-26482585/Quisinostat (QSS)^22^, could also induce RECK expression (Fig. 1g; Supplementary Table S3), decrease cell number (Fig. 1g), and induce flat reversion (Fig. 1h). The ortho-methyl isoform of DSK638, DSK637 (Fig. 1f, formula 1), showed little activity (Fig. 1b, e, g-i). Overall, the different HDAC inhibitors that induced RECK expression had similar effects on transformed cells in vitro.

### Suppression of metastasis by DSK638

**Figure 2:**
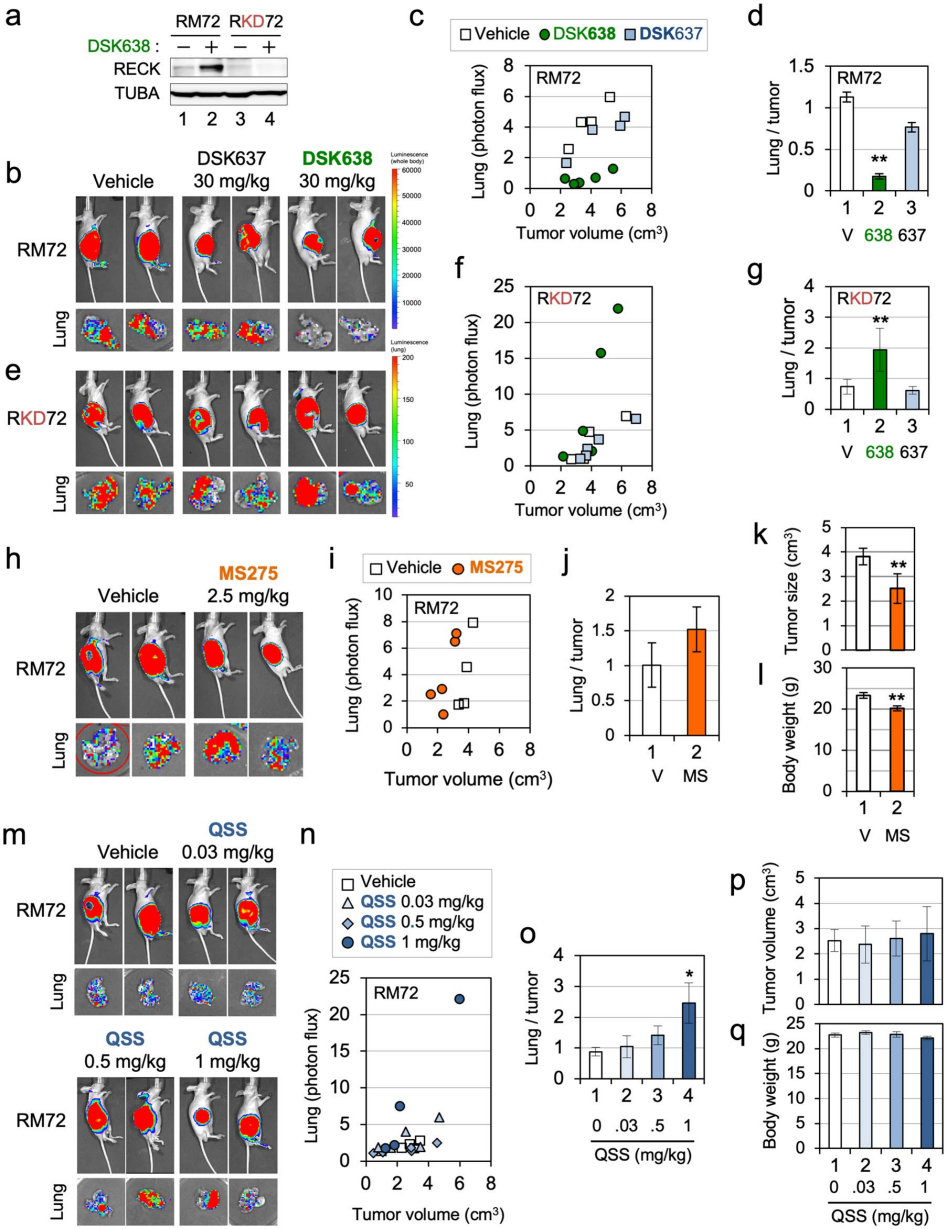
Metastasis-suppressing activity of DSK638. (**a**) Effects of DSK638 on the level of RECK protein in RM72 and RKD72 (RECK-depleted) cells. The cells were exposed to medium containing 10 µM DSK638 for 48 h, and the lysates were subjected to immunoblot assay. Note that DSK638 fails to induce RECK expression in RKD72 cells (lane 4). (**b-d**) Effects of DSK637 and DSK638 on the growth and lung metastasis of RM72 cells inoculated subcutaneously into nude mice. (**b**) The IVIS images of two typical cases per group after 2-weeks treatment with the indicated compound at the indicated dose. Upper panel: whole body. Lower panel: resected lung. (**c**) Relationship between tumor volume and lung metastasis (relative bioluminescence intensity; each dot represents one animal). (**d**) The ratio between lung metastasis and tumor volume. Bar represents mean ± s.e.m. Note the activity of DSK638 (data in green) to suppress lung metastasis of RM72 cells. (**e–g**) Effects of DSK637 and DSK638 on the growth and lung metastasis of RECK-depleted RKD72 cells. Experiments similar to those shown in b-d were performed using RKD72 cells. Note that DSK638 (data in green) failed to suppress lung metastasis of RKD72 cells. (**h–l**) Effects of MS275 on the growth and lung metastasis of RM72 cells inoculated subcutaneously into nude mice. Experiments similar to those shown in b-d were performed using MS275 (2.5 mg/kg). The effects on tumor sizes (k) and body weights (l) are also shown. Note that MS275 (data in orange) failed to suppress lung metastasis of RM72 cells at a dosage where tumor growth and body weight were slightly reduced. (**m-q**) Effects of QSS on the growth and lung metastasis of RM72 cells inoculated subcutaneously into nude mice. Experiments similar to those shown in h–l were performed using QSS at three doses (0.03, 0.5, and 1 mg/kg). Note that QSS (data in dark blue) stimulated lung metastasis of RM72 cells at a dosage (1 mg/kg) where tumor growth and body weight were unaffected.

DSK638 induced RECK expression in a luciferase-tagged, highly metastatic variant of HT1080, RM72^15^ (Fig. 2a, lane 2), but not in its RECK-depleted derivative, RKD72 (Fig. 2a, lane 4). Importantly, DSK638 suppressed lung-metastasis of RM72 cells (Fig. 2b-d) but not RKD72 cells (Fig. 2e-g), demonstrating that RECK is required for DSK638-mediated suppression of metastasis. DSK637 showed little metastasis-suppressing activity (Fig. 2b-d).

MS275 failed to suppress metastasis of RM72 (Fig. 2h–j) even at a dosage where tumor volume and body weight were slightly reduced (Fig. 2k,l), and QSS stimulated metastasis (Fig. 2m-o) at a dosage where tumor volume and body weight were unaffected (1 mg/kg; Fig. 2p,q). Thus, the ability to induce *RECK*-expression and flat reversion in vitro are not sufficient for suppression of metastasis in vivo.

### Transcription factors involved in the DSK638-response

**Figure 3:**
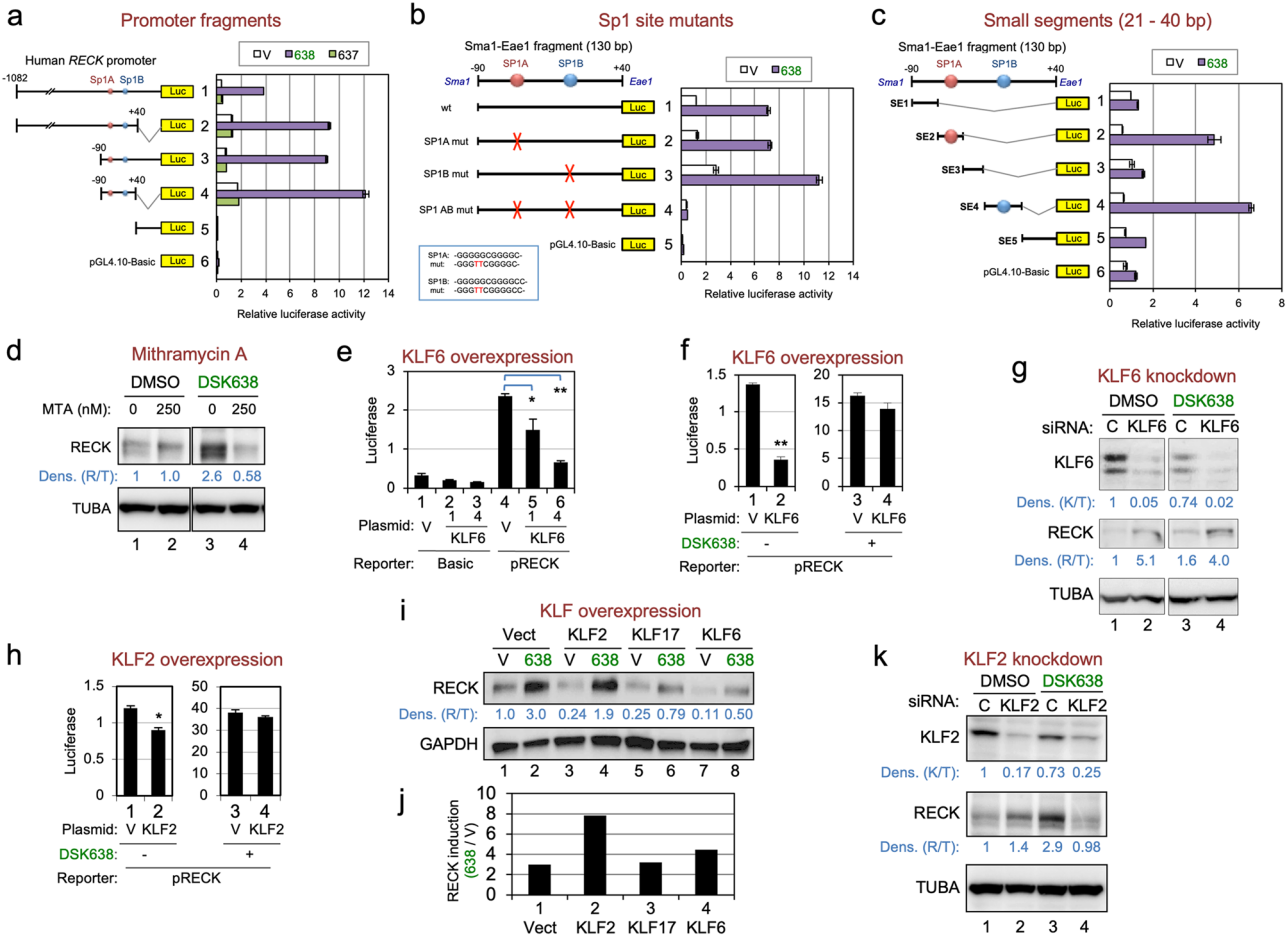
Mechanism of *RECK* gene activation by DSK638. (**a**) Location of DSK638-response element(s) in the *RECK* promoter. HT1080 cells were transiently transfected with a series of pGL4.10 plasmids containing various *RECK* promoter fragments inserted upstream of a firefly luciferase gene (fragments 1–5) or the vacant vector (No. 6), incubated in medium containing vehicle (white bar), DSK638 (10 µM; purple bar), or DSK637 (10 µM; green bar) for 24 h, and the luciferase activities in the cell lysates were determined. The data (mean ± s.e.m, n = 2) are presented as the ratios to the vehicle (DMSO)-treated controls. Positions of two Sp1 sites (Sp1A, Sp1B) are shown in the left diagram. Note that deletion mutant 4, the 130-bp Sma1-Eae1 fragment (SE130), showed robust promoter activity in the presence of 10 µM DSK638 (bar 4, compare purple bar with white and green bars). (**b**) Promoter activity and DSK638-response of SE130 without or with Sp1 site mutation(s). Red crosses in the left diagrams indicate the positions of Sp1 site mutations (base substitutions shown in red in the bottom box) in the SE130 fragments used in the luciferase assays. Note that SE130 carrying mutations in both Sp1 sites showed no response to 10 µM DSK638 (bar 4). (**c**) Promoter activity and DSK638-response of small sub-fragments of SE130. Note that the fragments containing only one Sp1 site were active in the presence of 10 µM DSK638 (purple bars 2 and 4). (**d**) Effects of Mithramycin-A on the expression of endogenous RECK protein in HT1080 cells. HT1080 cells were incubated for 24 h in medium containing vehicle (-) or medium containing DSK638 (10 µM), Mithramycin A (250 nM), or both. Cells were then lysed and subjected to immunoblot assay using anti-RECK (top panel) and anti-$\upalpha$-tubulin (internal control; bottom panel) antibodies. The relative density of the RECK band normalized against $\upalpha$-tubulin [Dens. (R/T)] is given below the top panel. (**e**) Effects of KLF6-overexpression on *RECK* promoter activity. HT1080 cells were co-transfected with pGL4.10 plasmid without any insert (Basic) or with the 1173-bp RECK promoter fragment (pRECK) and either empty expression vector (V), KLF6-expression vector at 0.1 µg/well, or KLF6-expression vector at 0.4 µg/well in a 12-well plate. After incubation for 24 h, the cells were lysed and subjected to luciferase assay. Note the dose-dependent effect of KLF6 on the reduction of *RECK*-promoter activity (bars 4–6). (**f**) Effects of KLF6-overexpression on the response of the *RECK* promoter to DSK638. HT1080 cells co-transfected with the pRECK reporter plasmid and the control vector (V) or the KLF6-expression vector (KLF6). Cells were incubated in medium containing vehicle (638: -) or 10 µM DSK638 (638: +) for 24 h, and the cells were then lysed and subjected to luciferase assay. The data are shown in two bar graphs with different Y-axis scale ranges. Note that KLF6 significantly reduced *RECK*-promoter activity in control medium (bars 2 vs. 1) but had little effect in the presence of DSK638 (bars 4 vs. 3). (**g**) Effects of KLF6-depletion on the level of RECK protein. HT1080 cells transfected with control siRNA (C) or siRNA targeting KLF6 were incubated for 48 h in medium containing vehicle (638: -) or 10 µM DSK638 (638: +). The cells were then lysed and subjected to immunoblot assay using anti-KLF6 (top), anti-RECK (middle), and anti-$\upalpha$-tubulin antibodies (bottom). The relative density of the KLF6 and RECK bands normalized against $\upalpha$-tubulin [Dens. (K/T) and Dens. (R/T)] is given below each panel. Note that the knockdown of KLF6 resulted in increased RECK band density both in the absence (lanes 2 vs. 1) and presence (lanes 4 vs. 3) of DSK638. (**h**) Effects of KLF2-overexpression on the response of the *RECK* promoter to DSK638. Experiments similar to those shown in f were performed using a KLF2-expression vector. Note that KLF2 reduces *RECK*-promoter activity (bars 7 vs. 5) but has little effect in the presence of DSK638 (bars 8 vs. 6). (**i, j**) Effects of overexpression of KLF2, KLF17, and KLF6 on the level of RECK protein. (**i**) HT1080 cells transfected with the vector expressing the indicated protein were incubated for 30 h in medium containing vehicle (638: -) or 10 µM DSK638 (638: +). The cells were then lysed and subjected to immunoblot assay using anti-RECK (top) and anti-GAPDH antibodies (bottom). Relative density of the RECK band normalized against GAPDH [Dens. (R/G)] is given below the top panel. (**j**) The ratio of normalized RECK band density of DSK638-treated cells to vehicle-treated cells is shown. Note that the highest induction ratio of RECK expression by DSK638 was achieved in the cells overexpressing KLF2 (bar 2). (**k**) Effects of KLF2-depletion on the level of RECK protein. HT1080 transfected with control or KLF2 siRNA were incubated for 48 h in medium containing vehicle or 10 µM DSK638. The cells were then lysed and subjected to immunoblot assay using antibodies against KLF2 (top panel), RECK (middle panel), and $\upalpha$-tubulin (bottom panel; internal control). Relative band density of KLF2 and RECK normalized against $\upalpha$-tubulin [Dens. (K/T) and Dens. (R/T)] is given below each panel. Note that DSK638 failed to upregulate RECK in KLF2-depleted cells (lane 4). Error bar in bar graphs: s.e.m. (n = 2). *p < 0.05, **p < 0.01 (Student’s t-test).

Luciferase reporter assays with a series of *RECK* promoter fragments revealed that the 130-bp Sma1-Eae1 fragment (designated SE130; Fig. 3a, fragment 4) exhibits a robust DSK638-response. SE130 contains two Sp1 sites (SP1A, SP1B) previously implicated in *RAS-*induced repression of *RECK* expression^23^. When both Sp1 sites in SE130 were mutated, DSK638-response was lost (Fig. 3b, purple bar 4). When SE130 was divided into five sub-fragments (SE1 to SE5; Supplementary Fig. S1), two fragments, each containing one Sp1 site (SE2 and SE4; see Supplementary Fig. S1), showed a robust DSK638-response (Fig. 3c, purple bars 2, 4). Mithramycin A, a compound that selectively binds GC-rich DNA^24,25^, suppressed induction of *RECK* expression by DSK638 (Fig. 3d, lane 4). These results indicate that the SP1 sites are involved in the DSK638-response.

Sp1 sites are targets of Sp1/KLF-family transcription factors: Kruppel-like factors (KLFs) recruit transcriptional regulatory proteins, including co-activators and co-repressors, to the promoter^26^. *KLF6,* a *KLF* family member, is abundantly expressed in RM72 cells (Supplementary Fig. S2a). Overexpression of *KLF6* suppressed *RECK*-promoter activity (Fig. 3e, bars 5, 6 vs. 4; Fig. 3f, bars 2 vs. 1), but suppression of RECK expression was abolished by DSK638 (Fig. 3f, bars 4 vs. 3). Depletion of KLF6 increased RECK protein expression both in the absence (5.1-fold; Fig. 3g, lanes 2 vs. 1) and presence (2.5-fold; Fig. 3g, lanes 4 vs 3) of DSK638. These results indicate that KLF6 represses the *RECK* promoter.

Another member of this family, *KLF2,* is strongly downregulated by DSK638 (Supplementary Fig. 2S). Overexpression of KLF2 suppressed *RECK*-promoter activity slightly (Fig. 3h, bars 2 vs. 1), but this suppression was abolished by DSK638 (Fig. 3h, bars 4 vs. 3). At the protein level, KLF2-overexpression significantly reduced the level of RECK (Fig. 3i, lanes 3 vs. 1), but this effect was less in the presence of DSK638 (Fig. 3i, lanes 4 vs. 2). The extent of RECK induction by DSK638 was higher in KLF2-overexpressing cells than in control cells or in cells overexpressing KLF17 (another abundantly expressed KLF) or KLF6 (Fig. 3j). Importantly, KLF2-depletion abolished the capability of DSK638 to increase RECK expression (Fig. 3k, lane 4). These results implicate KLF2 in *RECK*-upregulation induced by DSK638.

### Differential effects of HDAC inhibitors on RM72 cells in suspension

**Figure 4:**
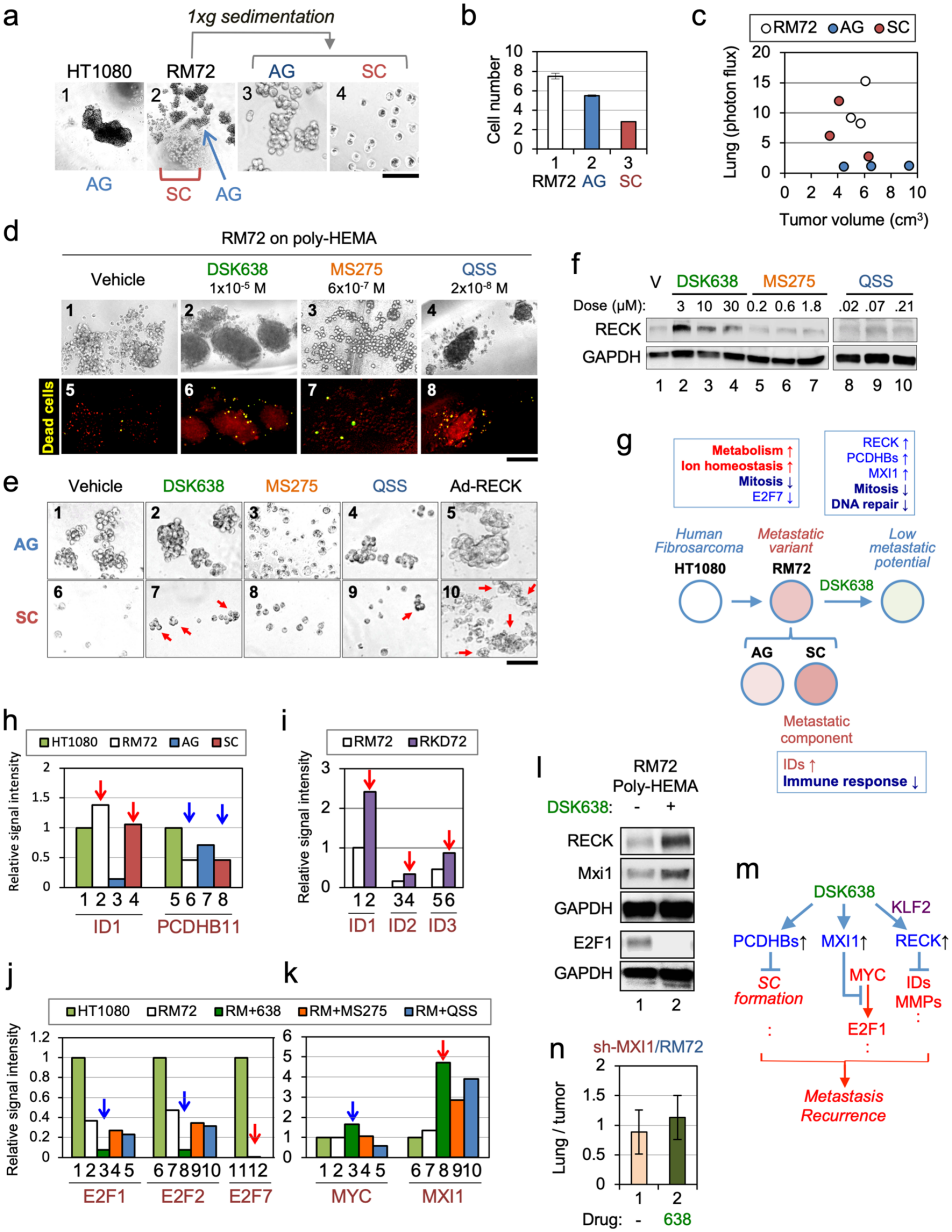
Mechanisms of metastatic conversion and metastasis-suppression by DSK638. (**a**) Heterogeneity of RM72 cells cultured in suspension. Phase contrast micrographs of HT1080 cells (1) and RM72 cells (2) incubated for 48 h on poly-HEMA-coated dishes. The RM72 cell suspension was then transferred to a 15-ml polypropylene tube with a wide-bored pipette and left in a vertical position for 5 min. The cells in the precipitate (3; enriched in AG) and supernatant (4; enriched in SC) fractions were separated and further incubated for 48 h on poly-HEMA-coated dishes. Scale bar: 500 µm for panels 1 and 2, 100 µm for panels 3 and 4. (**b**) Growth of AG and SC populations. RM72 cells or the fractionated AG and SC populations (1500 cells/well) were plated in triplicate onto 96-well tissue culture plates, and after 48-h incubation, the numbers of cells were determined. (**c**) Metastatic potential of AG and SC. RM72, AG, and SC were subjected to metastasis assays as described in Fig. 2. (**d**) Effects of three chemicals on the behaviors of RM72 cells cultured in suspension. RM72 cells plated onto poly-HEMA-coated 96-well plates (3000 cells/well) were incubated for 48 h in medium containing vehicle (DMSO), DSK638, MS275, or QSS at the indicated concentrations followed by Sytox Green staining. Scale bar: 200 µm. (**e**) Effects of three chemicals on AG and SC. Fractionated AG (1–4) and SC (6–9) were used in experiments similar to those shown in d. Cells infected with Ad-RECK were used as the control (5, 10). Scale bar: 100 µm. (**f**) Effects of three chemicals on the level of RECK in RM72 cells cultured in suspension. (**g**) Summary of our findings in transcriptome studies. The genes or events of prominent changes are shown in the boxes, in which upregulation and downregulation are indicated by upward and downward arrows. (**h–k**) Data of particular interest in our transcriptome studies. (**h**) Upregulation of *ID1* (red arrows) and downregulation of *PCDHB11* (blue arrows) in highly metastatic cell types (RM72 and SC). (**i**) Upregulation of *ID* family genes (red arrows) by RECK-knockdown. (**j**) Strong downregulation of *E2F1* and *E2F2* by DSK638 (blue arrows) and very low expression of *E2F7* (red arrow) in RM72 cells. (**k**) DSK638 upregulation of MYC (blue arrow) and MXI1 (red arrow). (**l**) Effects of DSK638 on the levels of RECK, MXI1, and E2F1 proteins in RM72 cells cultured in suspension. (**m**) A model of the molecular mechanisms of metastasis suppression by DSK638. (**n**) Suppression of MXI1 expression (Supplementary Fig. S8) makes RM72 cells refractory to the metastasis-suppressing activity of DSK638.

In suspension culture^27^, HT1080 cells form aggregates (Fig. 4a, panel 1) while the metastatic RM72 cell line produces two populations of cells: a single cell (SC) population and a population of aggregated cells (AG) (Fig. 4a, panel 2). When separated (Fig. 4a, panels 3 and 4), SC and AG populations proliferate more slowly than the mixed population of parental cells (Fig. 4b), suggesting metabolic cooperation between the two populations. Interestingly, AG populations have a lower metastatic potential than the parental RM72 cells or the SC population (Fig. 4c; Supplementary Fig. S3), suggesting that the SC population is the metastatic component of RM72.

When RM72 cells were treated with DSK638 in suspension culture, the size of the aggregates increased, the number of single cells decreased (Fig. 4d, panels 2 vs. 1), and some cells in the periphery of the aggregates died (Fig. 4d, panel 6). When treated with MS275, almost all RM72 cells were single (Fig. 4d, panel 3) and alive (Fig. 4d, panel 7) which was confirmed by flow cytometry (Supplementary Fig. S4). In contrast, numerous dead single cells were found after QSS-treatment (Fig. 4d, panel 8).

When RECK was overexpressed in separated populations, the AG population formed larger aggregates and the SC population formed small aggregates (Fig. 4e, panels 5 and 10). DSK638 induced similar changes in separated populations (Fig. 4e, panels 2 and 7). In contrast, MS275 caused aggregates to disassociate (Fig. 4e, panel 3). QSS had little effect on the AG population, but caused some aggregation of SC cells, although, QSS was less active than DSK638 in converting the SC population to an AG population (Fig. 4e, panels 4, 9 vs. 2, 7).

We also found that in suspension culture, RECK protein was robustly induced by DSK638 but not by MS275 or QSS (Fig. 4f). Such anchorage-independent action of DSK638 may also contribute to its unique bioactivity. Hence, RM72 in suspension culture responded differently to these three HDAC inhibitors.

### Molecular mechanisms of metastatic conversion and metastasis suppression

To gain insights into the molecular mechanisms of the phenotypic changes described above, we analyzed the transcriptomes of the following cell sets and their respective controls: (i) four cell types without treatment: HT1080 (control), RM72, AG, SC; (ii) RM72 cells treated with three drugs (DSK638, MS275, QSS); (iii) RM72 cells infected with RECK-adenovirus; and (iv) RECK-depleted RKD72 cells treated with DSK638. Gene Set Enrichment Analysis (GSEA)^28^ yielded three remarkable findings (summarized in Fig. 4g). First, the gene set NEGATIVE_REGULATION_OF_METABOLIC_PROCESS was enriched in the transcripts more abundant in the SC population than in the AG population (Supplementary Tables S4, S5); the leading-edge subset of this comparison contains three *ID*-family genes (*ID1, ID2, ID3*) encoding transcription factors associated with suppression of cell differentiation^29^. The levels of *ID1* in the RM72, SC, and AG populations parallel their metastatic potentials: high in RM72 and SC, and low in AG (Fig. 4h, bars 2–4; Fig. 4c). *ID1, ID2,* and *ID3* were upregulated in RECK-depleted cells (Fig. 4i), suggesting that RECK suppresses these genes.

Second, the gene set EXTRACELLULAR_STRUCTURE_ORGANIZATION_AND_BIOGENESIS was enriched in the transcripts increased by DSK638 (Supplementary Tables S6, S7); the leading-edge subset of this comparison contains eight *PCDHB*-family genes encoding neural cell-adhesion molecules^30–32^. In RM72 cells, *PCDHB11* is strongly upregulated by DSK638 (Supplementary Fig. S5a). The levels of *PCDHB11* in the RM72, SC, and AG populations show an inverse correlation with their metastatic potentials: lower in RM72 and SC, and higher in AG (Fig. 4h, bars 6–8; Fig. 4c). DSK638 upregulates *PCDHB11* in RECK-depleted RDK72 cells (Supplementary Fig. S5b, bar 4), and RECK-overexpression does not upregulate *PCDHB11* in RM72 cells (Supplementary Fig. S5b, bar 6), suggesting RECK-independence of *PCDHB11*-upregulation by DSK638. *PCDHB11*-overexpression in RM72 cells resulted in the formation of tighter aggregates (Supplementary Fig. S5c, panels 2 vs. 1) and in reduced metastatic potential (Supplementary Fig. S5d, bars 3 vs. 1). Hence, PCDHB proteins may contribute to the increased cell–cell adhesion (Fig. 4e, panels 2, 7) and metastasis-suppression (Fig. 2c, d) mediated by DSK638.

Third, the gene set MITOTIC_CELL_CYCLE was enriched in transcripts decreased by DSK638 (Supplementary Tables S8, S9) and in transcripts that were less abundant in RM72 than in HT1080 (Supplementary Tables S10, S11); the leading-edge subsets of these two comparisons largely overlapped, suggesting that DSK638 inhibits the mitotic cell cycle, a function already attenuated in RM72 (Supplementary Fig. S6). Of note, both leading-edge subsets contain *E2F1* (a proliferation-associated transcription factor activated by MYC^33^) and a number of E2F1-targets. We therefore focused on *E2F*-family and *MYC*-family genes (Supplementary Fig. S7) and found the following: (i) *E2F7,* encoding a “repressor E2F”^34^, is expressed in HT1080 but undetectable in RM72 (Fig. 4j, bars 11 vs. 12, red arrow; Supplementary Fig. S7a). (ii) *E2F1* and *E2F2* mRNAs are less abundant in RM72 than in HT1080 (Fig. 4j, bars 2, 7 vs. 1, 6) and are further downregulated by DSK638 (Fig. 4j, bars 3, 8 vs. 2, 7). Downregulation of *E2F1* and *E2F2* transcript levels by MS275 and QSS was less than that by DSK638 (Fig. 4j, bars 4, 5 vs. 3 and bars 9, 10 vs. 8). (iii) DSK638 does not downregulate *MYC* (Fig. 4k, bars 3 vs. 2). (iv) *MXI1*, a putative tumor suppressor encoding a MYC-antagonist^35,36^, was strongly upregulated by DSK638 (Fig. 4k, bars 8 vs. 7). Upregulation of *MXI1* by MS275 or QSS was less than by DSK638 (Fig. 4k, bars 9, 10 vs. 8). DSK638 upregulates *MXI1* in RECK-depleted RKD72 cells (Supplementary Fig. S7c, bars 1–4), and RECK-overexpression fails to upregulate *MXI1* (Supplementary Fig. S7c, bars 6 vs. 5), indicating RECK-independence of *MXI1*-upregulation by DSK638. (v) DSK638 upregulates *MXI1* and downregulates E2F1 at the protein level (Fig. 4l). These findings support the idea that DSK638 upregulates MXI1 which antagonizes MYC, leading to the downregulation of E2F1, E2F2, and their targets, resulting in suppression of mitotic cell cycle progression.

Taken together, our data indicate that DSK638 activates at least three molecular systems: RECK (a regulator of *ID* expression and extracellular proteases), PCDHBs (mediators of cell–cell adhesion), and MXI1 (an inhibitor of mitotic cell cycle progression) (Fig. 4m). Consistent with this model, DSK638 failed to suppress the metastasis of RM72 cells with reduced RECK expression (RKD72 in Fig. 2e, 2f, and 2g) or reduced MXI1 expression (compare Fig. 4n with Fig. 2d).

## Discussion

Compelling evidence implicates RECK in metastasis suppression^6,12–14,37,38^. Our screen for chemicals capable of inducing *RECK* expression and flat reversion in tumor cells led to the identification of a metastasis suppressing drug, DSK638. Intra-peritoneal injection of DSK638 suppresses lung-metastasis of a fibrosarcoma cell line, RM72, subcutaneously inoculated into nude mice. Our findings also shed light on mechanisms by which these sarcoma cells acquired metastatic potential and how DSK638 activates RECK expression and suppresses tumor metastasis.

E2F7 is a feedback regulator of E2F1 capable of inducing cell-cycle-arrest under the control of RB and p53^34,39^. The absence of E2F7-expression in RM72 cells is likely to contribute to their malignant properties. If so, however, the observed downregulation of E2F1/E2F2 and E2F1-targets in RM72 cells compared to the parental HT1080 cells (Fig. 4J, bars 2, 7; Supplementary Table S11) appears counterintuitive; it might reflect adaptation to the absence of the negative regulator E2F7 and/or their slow-cycling, stem-cell-like character.

Our data implicate the transcription factor KLF2 in *RECK*-upregulation by DSK638. DSK638 downregulates *KLF2* mRNA (Fig. S2a) and weakens KLF2 inhibition of RECK promoter activity (Fig. 3h), suggesting that reduction in KLF2-mediated transcriptional repression underlies DSK638-mediated *RECK* gene activation. This model was supported by the observation that in the cells overexpressing KLF2, the level of RECK protein was lower than the control when incubated in regular medium but higher in medium containing DSK638, making the ratio of induction greater (Fig. 3i). Unexpectedly, however, when KLF2 was depleted, the level of RECK protein was somewhat reduced in the presence of DSK638 (Fig. 3k, lane 4), as if under these conditions, KLF2 is required for RECK expression. A possible model to explain these findings is that DSK638 converts KLF2 from a repressor to an activator essential for DSK638-stimulated RECK-upregulation (Fig. S9a). Given that recruitment of transcriptional regulators by a closely related DNA-binding protein, KLF1, is switched by acetylation^40^ and that the relevant acetylation sites (K288 and K302 in KLF1) are conserved in KLF2 (Fig. S2b), it is tempting to speculate that DSK638, probably acting as an HDAC inhibitor (Table S2), might convert KLF2 from a repressor to an activator by inhibiting deacetylation of these sites.

*KLF2* is frequently mutated in splenic marginal zone lymphoma^39,41^, and downregulated in ovarian^41^ and prostate^42^ cancers. KLF2 inhibits invasion and/or metastasis of prostate^43^ and colon^44^ cancer cells. Our findings raise the possibility that at least a part of such anti-oncogenic functions of KLF2 may be mediated by RECK. In support of this premise, we found that RECK had a negative effect on *ID*-expression: in many cell types, ID proteins block differentiation, maintain stemness, and exerts oncogenic/pro-metastatic effects^29^. KLF2 is also known to play important roles in various physiological processes, including lung development^45^, cardiovascular development^46^, hematopoiesis^47^, immune cell differentiation/functioning^26,48–50^, and atheroprotection^26,51^. Possible roles for RECK as a conditionally regulatable effector of KLF2 in these processes should be interesting subjects for future studies.

MXI1 is a known MYC-antagonist^33,36^, and *MXI1*-upregulation is likely a critical event in metastasis-suppression by DSK638: *Mxi1*-deficient mice are hyperplastic in multiple tissues^33^, suggesting a role for MXI1 in tumor suppression. Among the three HDAC inhibitors examined, the strengths of MXI1 upregulation and E2F1/E2F2 downregulation are correlated (Fig. 4k, bars 8–10 vs. Figure 4j, bars 3–5, 8–10), supporting our model that MXI1 is involved in the E2F1-downregulation induced by DSK638 (Fig. 4m). Although the relevance of MXI1 to cancer metastasis remains unexplored, our data, together with the previous observations that MYC and E2F1 promote epithelial-mesenchymal transition and metastasis^52,53^, implicate MXI1 in metastasis-suppression.

DSK638 upregulates multiple *PCDHB*-family genes. Protocadherins (PCDHs) play important roles in specific cell–cell adhesions during neural development^30–32^. Anti-oncogenic functions of some PCDHs have also been reported, although, their mechanisms of action remain obscure^54^. Our data indicate that PCDHB11, a member strongly upregulated by DSK638 in RM72 cells, may contribute to metastasis-suppression by enhancing their cell–cell adhesion. Thus, DSK638 promotes two pathways that affect extracellular interactions: PCDHB11, as described here, and RECK, a membrane-anchored protein that regulates peri-cellular proteolysis^6–10,55^.

We found that the single cell population of RM72 cells had a higher metastatic potential than the aggregate population, although cell clustering has previously been implicated in breast cancer metastasis^56^. The apparent disparity may reflect the differences in experimental systems employed and/or the step(s) of metastasis being focused on.

Recent studies have yielded several important advances in understanding the role of RECK in angiogenesis. Characterization of tissue-selective knockout mice revealed that RECK expression in both endothelial cells and mural cells is required for embryonic vascular development and survival; in mural cells RECK is essential as early as the mid-gestation period and in later embryonic stages RECK is required in endothelial cells for proper brain angiogenesis^57^. Aortic ring assays revealed that *Reck*-deficiency resulted in the formation of excessive vascular sprouts that are prone to fuse with each other and are poorly associated with mural cells and the ECM^57^, implicating RECK in blood vessel maturation. Endothelial RECK was found to serve as a ligand-selecting component of the WNT7 receptor required for brain angiogenesis and blood–brain-barrier formation^58–61^. RECK in neural precursor cells, a WNT7-producer, was also found to be essential for forebrain angiogenesis, probably by facilitating the delivery of active ligand to the receptor expressed on the surface of endothelial cells^62^. Hence, RECK protein is expressed in multiple cell types where it probably has different functions but acts in concert to help coordinate proper vascular development.

On the other hand, forced RECK expression in cancer cells was found to result in suppression of tumor angiogenesis and metastasis in a mouse xenograft model^7^. Tumor vessels are often immature and poorly associated with mural cells and the ECM^63,64^. Overexpression of RECK in tumor cells results in a limited number of large vessels well-covered by basement membrane^7^, suggesting that RECK expression in the surrounding tissues (i.e., tumor cells) suppresses the branching of tumor vessels derived from the host animal. Thus, RECK promotes normal angiogenesis but suppresses tumor angiogenesis.

Although the exact mechanism(s) by which RECK suppresses tumor metastasis remains unclear, downregulation of *ID* (this study) and normalization of tumor vessels (see above) may contribute to this suppression. Other RECK-mediated processes relevant to tumor metastasis include the regulation of ECM-degradation^65^, Notch-signaling^9^, STAT3-signaling^66^, cell migration^67^, mesenchymal phenotype^68^, fibrillin fiber formation^55^, and cellular senescence^69^. In addition to these RECK-mediated events, DSK638 may also affect other processes such as regulation of cell cycle progression (via MXI1) and cell–cell adhesion (via PCDHBs). Consequently, RECK may be considered a useful marker as well as an effector for drug screening (Fig. S9b). This study has also demonstrated the practical value of our approach using a molecular marker (e.g., RECK) for a primary screen and multiple phenotypic assays for secondary screening.

Since RM72 may represent only a part of the metastatic properties of malignant cancers^4^, testing DSK638 in other metastasis assay systems would be an important next step in developing metastasis-suppressing drugs of clinical value^5^. Such drugs, once proven to have reasonably low side effects, may be useful for preventing or retarding recurrence in cancer patients. Drugs that prevent metastatic colonization may be useful for adjuvant chemotherapy. We expect that drugs that prevent steps after metastatic colonization should have wide therapeutic applications. Continued efforts to develop bioassay systems that represent defined step(s) of metastasis will be of great benefit in achieving this goal.

## Methods

### Chemicals

The DSK compounds were synthesized by SUMIKA TECHNOSERVICE (Osaka): the DSK Project was a joint research project between Kyoto University and Dainippon Sumitomo Pharma, Co., Ltd. Sources and catalogue numbers of the other chemicals are listed in Supplementary Table 1. For in vitro use, these chemicals were dissolved in DMSO (Sigma Aldrich) to a concentration of 10 mM, stored at -20˚C, and further diluted to an appropriate final concentration in appropriate medium upon use. For in vivo studies, chemicals were dissolved in olive oil and administrated daily by intraperitoneal (ip) injection for 14 days.

### Drug screening

Details of high throughput screening will be described elsewhere (manuscript in preparation). Briefly, a 1-kb RECK promoter fragment was inserted into the multicloning sites of the pL4.10 vector (Promega) to generate pL4.10-phRECK. HT1080 cells plated on the previous day were transfected with a mixture of two plasmids, pL4.10-phRECK and pTK-RLuc (Renilla luciferase reporter vector). After 6 h, the cells were exposed to the test chemical (3 µM) for 24 h and then subjected to a dual luciferase assay to identify compounds that activate the RECK promoter. The 43 compounds that gave rise to more than twofold increase in luciferase activity by pL4.10-phRECK were examined further using immunoblot assays.

### Immunoblot assay

HT1080 cells plated on the previous day were treated with the test chemical or the vehicle (DMSO) in growth media for 48 h. The cells were lysed as described previously^69^. The protein extracts were separated by electrophoresis on a 10% SDS polyacrylamide *gel*. Protein detection was performed using the antibodies described in Supplementary Table S1. For visualization, the Enhanced Chemiluminescence kit (Millipore) was used. Images were recorded and analyzed using LAS-4000 and the MultiGauge software (Millipore) according to the manufacturer’s instructions.

### Quantitative flat reversion (qRev) assay

DT cells were stably transfected with pmCherry (Clontech 632,522), and a clone (named DSK4b) uniformly emitting bright red fluorescence was isolated. DSK4b cells (2,000 cell/well) were seeded onto 96-well tissue culture plates, and test chemicals (in serial dilutions) were added at 8 h. After incubation for 72 or 96 h, the nuclei were stained with Hoechst33342 (0.5 µg/ml) just before image analysis. The number of nuclei per unit area (blue fluorescence; n) and the total area of red fluorescence per unit area (A) were recorded using a cell image analyzer (Cytell, GE or ArrayScan VTI, Thermo Fischer). The area per cell (A/n) represents the activity of a drug to induce flat reversion, and the number of nuclei (n) is related to the cytotoxicity of the test chemical.

### Matrigel invasion assay

HT1080 cells plated on the previous day were treated with the test chemical (10 µM) or the vehicle (DMSO) in growth media for 48 h. FluoroBlok Transwell Inserts (8 -mm pore size; Corning) were pre-coated by adding 100 µl diluted Matrigel (BD Biosciences; 25 µg/100 µl) onto the membranes and air drying overnight. The coated inserts were placed into 24-well plates containing growth media as a chemo-attractant. The cells treated with the test chemical were pre-labeled for 30 min with CellTracker Green CMFDA (Life Technologies) and suspended in DMEM containing 0.1% FBS and the test chemical and plated onto the insert. After 24-h incubation, the cells that had invaded to the lower side of the membrane were photographed (4 fields/2 inserts/sample) using an inverted fluorescent microscope (KEYENCE BZ-9000). The area occupied by the fluorescent, invading cells was quantified using Image J.

### Gelatin zymography

RM72 cells plated on the previous day were treated with the test chemical (10 µM) or the vehicle (DMSO) in growth media for 24 h. The cells were then exposed to serum-free DMEM for 24 h, and the supernatants were subjected to electrophoresis on a 1% SDS–polyacrylamide gel containing 0.1% gelatin under non-reducing conditions. The gel was washed twice in 2.5% Triton X-100, 10 mM Tris–HCl pH 8.0 for 30 min and once in 10 mM Tris–HCl pH 8.0 for 30 min at room temperature. The gel was then incubated in 50 mM Tris–HCl (pH 8.0), 0.5 mM CaCl_2_, and 1 µM ZnCl_2_ for 24 h at 37˚C followed by staining with Coomassie Brilliant Blue G-250.

### Cell culture

The human fibrosarcoma cell line HT1080 (CCL-121, ATCC) was maintained in Dulbecco’s modified Eagle (DME) medium supplemented with 10% fetal bovine serum and penicillin–streptomycin (GM). RM72, a derivative of HT1080 carrying a luciferase gene and recovered from a lung metastasis after subcutaneous inoculation into a nude mouse^15^ was maintained in GM containing 400 µg/ml hygromycin-B (Roche). To obtain RKD72, RM72 cells were infected with a lentiviral vector expressing shRNAs targeting RECK (Thermo Scientific Open Biosystems) and selected in GM containing 4 µg/ml puromycin (Sigma Aldrich). Stable and transient transfection of plasmid DNA was performed using Lipofectamine 2000 (Thermo Fisher Scientific). siRNA transfection was performed using RNAiMax (Thermo Fisher Scientific). Plasmid vectors expressing KLF2 (SC127849), KLF6 (SC119291), and KLF17 (SC324238) were obtained from OriGene. Silencer Select RNAi for KLF2 (4,392,420), KLF6 (AM16708), and the negative control were obtained from Thermo Fisher Scientific. For suspension culture, plastic tissue-culture dishes coated with poly-HEMA [poly (2-hydroxyethyl methacrylate)] (Sigma Aldrich) were used^27^. To prepare AG and SC populations, RM72 cells incubated on poly-HEMA-coated dishes (2 × 10^5^cells/60-mm dish) for 72 h were gently transferred using a wide-bored pipette into a 15-ml polypropylene tube (2325–015、IWAKI), and the tube was left in vertical position for 5 min, followed by careful recovery, using pipettes, of the supernatant (SC) and sedimented (AG) fractions. Dead cells were stained using SYTOX Green (Invitrogen).

### COMPARE analysis

Relative sensitivity of a panel of 39 human cancer cell lines to growth inhibition by DSK638 was determined following the standard protocol^18,70^. The fingerprint of responses (i.e., a set of thirty nine GI_50_ values) of DSK638 was compared to those of the other compounds in the database using the COMPARE algorithm^19^ which gives rise to the Pearson correlation coefficient (*r*) between DSK638 and the respective test compounds (see Supplementary Table S2 for the top 20 drugs that share a similar inhibitory spectrum with DSK638).

### Spontaneous tumor metastasis model in nude mice

The experiments using mice were approved by the Animal Research Committee, Kyoto University, and were performed in accordance with MEXT Notice No. 71 and the Act on Welfare and Management of Animals, Japan; the present report was prepared in compliance with the ARRIVE guideline. Spontaneous metastasis assays were performed as previously described^15^. In brief, RM72 cells or RKD72 cells (3 × 10^6^) suspended in 0.1 ml PBS were injected subcutaneously into the right posterior flank of Balb/c nu/nu mice (6 weeks old, male, Charles River). Small tumors (~ 3 × 3-mm diameter) developed 5 days after injection. The mice were randomly divided into groups (n ≥ 5 animals per group) and treated with vehicle or the test chemical via intra-peritoneal injection. After 14-d treatment, the fates of tumor cells in the mice were assessed by bio-imaging (see next section). The tumor volume (length x width x height) and body weight were measured once a week. The sample size of at least five animals (with visible tumors at day 5 after cell inoculation) per group was chosen to make experiments manageable (especially, the daily treatment with drugs) and to obtain reproducible results based on our experience with this assay. The animals with no visible tumor at day 5 were excluded from the experiments. Data for all individual animals were plotted on a graph (X-axis: tumor volume; Y-axis: photon flux from resected lung). All cages were kept under comparable conditions. The order of treatments and measurements were not randomized, but all data retrospectively confirmed that the observed differences could not be explained by such artefacts. Animal experiments were carried out by Y.Y., K.Y., and M.N.; measurements were not blinded, but all animal experiments were not hypothesis-driven and performed objectively.

### Tumor imaging in vivo

Mice were anesthetized and injected intraperitoneally with 75 mg/kg of d-luciferin (Promega) in PBS (-). Bioluminescence images were acquired with the IVIS Imaging System (Xenogen) at 5 min after injection. Photons emitted from living mice or isolated organs were measured with a recording period of 60 s using the Living Image software (Xenogen).

### Reporter construction

Promoter deletion mutants shown in Fig. 3a were generated as follows. No. 3 (-177 to -1): pL4.10-phRECK was digested with XhoI and SmaI, and the cohesive end was filled in followed by self-ligation (i.e., circularization). No. 2 (-1173 to -48) and No. 5 (-48 to -1): The 1.3-kb KpnI-PvuII fragment from pL4.10-phREC was digested with EaeI to isolate the KpnI-EaeI (1.14 kb) and EaeI-PvuII (0.16 kb) fragments whose cohesive ends were then filled in. The KpnI-EaeI (1.14 kb) fragment was ligated into pL4.10 digested with Kpn I and EcoRV to obtain construct No. 2. The EaeI-PvuII (0.16 kb) fragment was further digested with HindIII and ligated into pL4.10 digested with EcoRV and Hind III to obtain construct No. 5. No. 4 (-177 to -48): Construct No. 2 was digested with Xho I and Sma I, and the cohesive end was filled in followed by self-ligation (i.e., circularization). The Sp1 site mutants shown in Fig. 3b were generated using QuikChange Lighting Site-Directed Mutagenesis Kit (Agilent). For the small segments of RECK promoter tested in Fig. 3c (sequences are shown in Supplementary Fig. S1), annealed synthetic oligonucleotides were cloned into pGL4.10.

### Luciferase reporter assay

In experiments with DSK638 a duel luciferase assay using firefly luciferase as the reported vector and the *Renilla* luciferase vector (pRL-TK) as the control vector could not be used: DSK638 activated *Renilla* luciferase activity from pRL-TK to such an extent that it could not serve as an internal control. Therefore, in experiments with DSK638 firefly luciferase activity alone was measured. HT1080 cells (40,000 cells/well) plated into 12-well plates on the previous day were co-transfected with the promoter-reporter constructs using Lipofectamine 2000. After incubation for 24 h, the cells were treated with vehicle or the test chemical for 24 h. Luciferase activity was measured using the Dual-Glo Luciferase Assay System (Promega).

### Gene expression profiling

The cells were incubated under appropriate conditions for 30 h. Total RNA was extracted from these cells using an RNeasy Mini Kit (Qiagen, Hilden, Germany) and subjected to transcriptome assay using SurePring G3 Human GE 8 × 60 K Microarray (Agilent). The data were processed with the robust multiarray average algorithm^71^ using GeneSpring and subjected to gene set enrichment analysis (GSEA; Broad Institute)^28^ in April 2017.

### Quantitative reverse transcription-polymerase chain reaction (qRT-PCR).

qRT-PCR was performed as previously described^68^. Briefly, total RNA was extracted using an RNeasy Mini Kit (QIAGEN). The levels of specific mRNAs were determined using SuperScript III Platinum SYBR Green One-Step qRT-PCR Kit (Invitrogen) with the Mx 3005P Real-Time PCR System and Mx Pro software (Stratagene). Primers for human RECK were 5’-GCTGGCAATTTGGTGTGCTCTA-3’ and 5’-GGGTAAGTGCGCCCATTCTG-3’. Primers for the control human hypoxanthine phosphoribosyl-transferase 1 (HPRT1) were 5’-CCAGACAAGTTTGTTGTAGG-3’ and 5’-TCCAAACTCAACTTGAACTC-3’. The reaction consisted of an initial reverse transcription [50 °C, 5 min; 95 °C, 5 min] followed by 45 cycles of PCR [94 °C, 15 s; 54 °C (HPRT1) or 64 °C (hRECK), 40 s; 72 °C, 20 s].

### Cell cycle analysis

Cell cycle analysis was performed as described previously^69^. Briefly, cells incubated for 30 h under appropriate conditions were fixed for 20 min in 1% methanol in PBS (-) on ice and then stained in PBS (-) containing 20 µg/ml propidium iodide and 250 µg/ml DNase-free RNaseA for 30 min at room temperature. The stained cells were analyzed by flow cytometry using FACSVerse (BD Biosciences) with the CELLQuest software.

### Statistical analyses

Statistical significance was assessed by Student’s t-test using the Excel software; F-test (Excel) was used to assess whether the variances are equal or unequal.

## Supplementary Information

Supplementary Information 1.

Supplementary Information 2.
