# Supplementary material for: Suppression of tumor metastasis by a RECK-activating small molecule
Source: Sci Rep. 2022 Feb 11;12:2319. doi: 10.1038/s41598-022-06288-3 (PMC8837781; doi:10.1038/s41598-022-06288-3)
Supplement: Supplementary file 1 — Supplementary Information 1. [file 41598_2022_6288_MOESM1_ESM.pdf]

Suppression of tumor metastasis by a *RECK*-activating small molecule

Yoko Yoshida, Kanako Yuki, Shingo Dan, Kanami Yamazaki, and Makoto Noda

Supplementary Figures

```
                                      SmaI-EaeI fragment (130 bp)
GGGAGGTTTT GGAAACACTG TGAGGCAGGG GGCGGGGCTT GAGCGGGCCG CAGCCAGTCA CCAAAGGGCC GGGCGCTGGG GGCGGGGCCT CGCGCGAGCG GCGGCGGTAG CGGCGGCAGC GGCTGCGGCC

GGGAGGTTTT GGAAACACTG TGA
    SE1 (23 bp)        GAGGCAGGG GGCGGGGCTT G
                            SE2 (20 bp)      GAGCGGGCCG CAGCCAGTCA CCAAAG
                                                     SE3(26 bp)        AGGGCC GGGCGCTGGG GGCGGGGCCT CGCGCGA
                                                                                   SE4(33 bp)         CGAGCG GCGGCGGTAG CGGCGGCAGC GGCTGCGGCC
                                                                                                                SE5(36 bp)
```

Supplementary Figure S1. Small segments of the *RECK* promoter used in the experiments shown in Fig. 3c. The two Sp1 sites are highlighted in red.

a

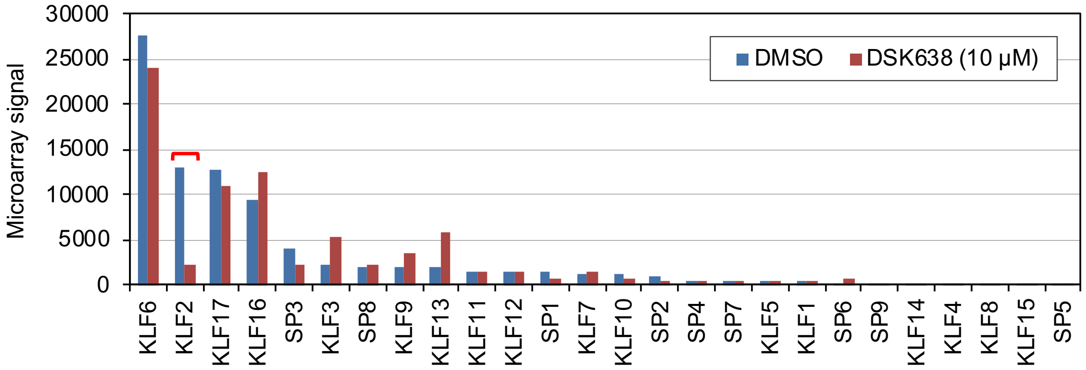

b

```
                          Transactivation   Repression
                           (CBP-binding)   (Sin3A-binding)
                                       |      |
KLF1   274  KRQAAHTCAHPGCGKSYTKSSHLKAHLRTHTGEKPYACTWEGCGWRFARSDELTRHYRKH   333
KLF2   267  KRTATHTCSYAGCGKTYTKSSHLKAHLRTHTGEKPYHCNWDGCGWKFARSDELTRHYRKH   326
```

Supplementary Figure S2. Expression and structural features of KLF2. (**a**) Expression of *KLF/Sp1* family transcription factor genes in RM72 cells and the effects of DSK638 as assessed from transcriptome data. RM72 cells were incubated for 30 h on poly-HEMA-coated dishes in medium containing vehicle (blue bars) or 10 μM DSK638 (red bars). Note the strong downregulation of *KLF2* (red bracket). (**b**) Alignment of KLF1 and KLF2 amino acid sequences. Note that the two lysine residues (K) highlighted in red and blue are conserved between the two proteins.

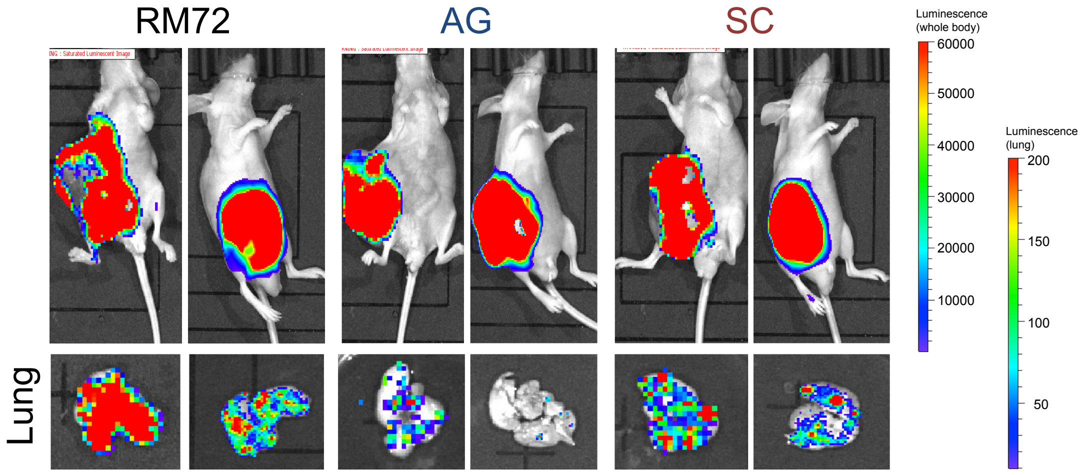

Supplementary Figure S3. Different metastatic potentials of RM72 subpopulations, AG and SC. Examples of *in vivo* imaging data that are summarized in Fig. 4c are shown. Compare the signals in the resected lung tissues (bottom panels).

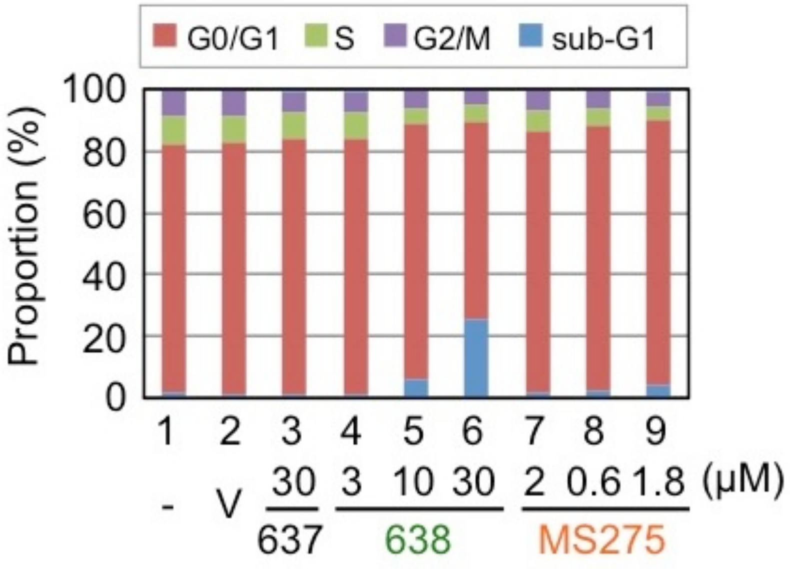

Supplementary Figure S4. Cell cycle analysis of RM72 cells incubated for 30 h on poly-HEMA coated dishes in medium containing DSK637, DSK638, or MS279 at the indicated concentrations. Note the clear increase in the sub-G1 population (blue zone) after the treatment with DSK638.

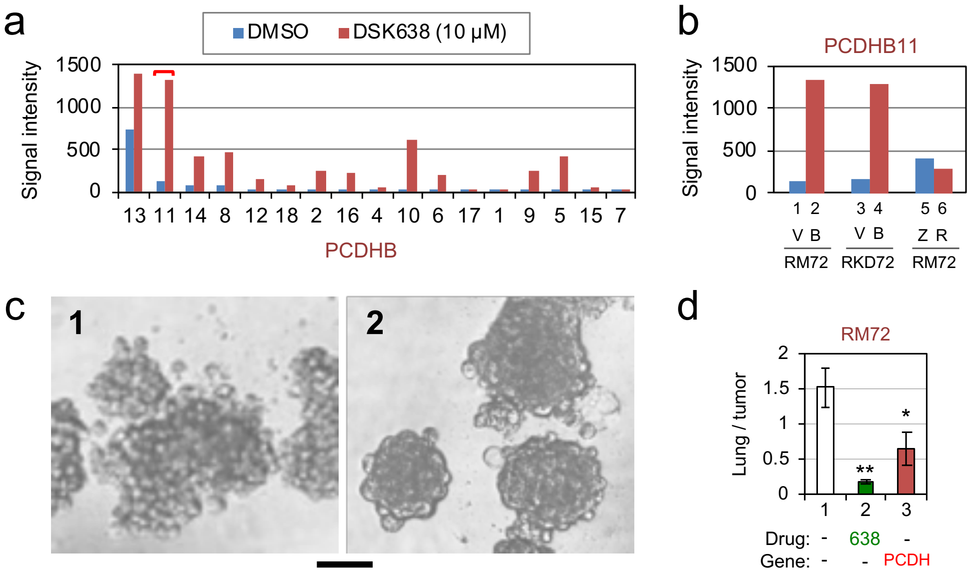

Supplementary Figure S5. Expression and activity of *PCDHB11*. (**a**) Expression of *PCDHB* family genes in RM72 cells and the effects of DSK638 as assessed from transcriptome data. RM72 cells were incubated for 30 h on poly-HEMA-coated dishes in medium containing vehicle (blue bars) or 10 μM DSK638 (red bars). Numbers below the horizontal axis correspond to the numerical part of the *PCDHB* gene name. Note the strong upregulation of *PCDHB11* (red bracket). (**b**) Effects of DSK638 and RECK on *PCDHB11* expression. Signal intensity of *PCDHB11* in the transcriptome data for RM72 cells (lanes 1, 2, 5, 6) or RKD72 cells (RECK-depleted RM72 cells; lanes 3, 4) treated with vehicle (V), DSK638 (D), control adenovirus (Z), or RECK-expressing adenovirus (R) is shown. Note that DSK638 upregulates *PCDHB11* even when RECK is depleted (lane 4) and that RECK overexpression fails to upregulate *PCDHB11*. (**c**) Effects of PCDHB11 on spheroid formation of RM72 cells. RM72 cells stably transfected with a vacant vector (panel 1) or a *PCDHB11*-expression vector (panel 2) incubated for 30 h on poly-HEMA-coated dishes were observed under a phase-contrast microscope. Scale bar: 100 μm. (**d**) Effects of *PCDHB11* overexpression on the metastatic potential of RM72 cells. RM72 cells stably transfected with a vacant vector (panel c1) or a *PCDHB11*-expression vector (panel c2) were subjected to metastasis assays as described in Fig. 2. Mice inoculated with RM72 cells stably transfected with the vacant vector were treated without (bar 1) or with (bar 2) DSK638.

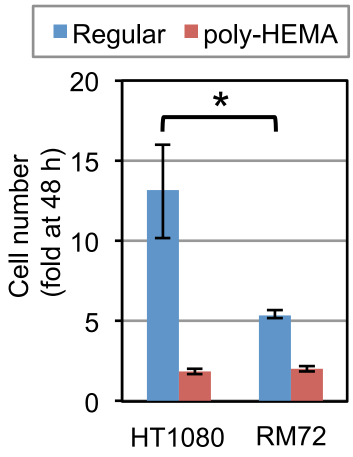

Supplementary Figure S6. Growth of HT1080 and its metastatic variant RM72 on standard or poly-HEMA-coated plates. Cells (1500/well) were incubated for 24 h or 72 h on standard 96-well tissue culture plates or poly-HEMA-coated plates. Cell number was determined using Cell Count Reagent SF (Nacalai Tesque). Bars represent the ratio between the values at 72 h and 24 h (mean ± sem) of triplicate samples. *$P<0.05$. Note that RM72 cells proliferate more slowly than the parental HT1080 cells on standard plates, while there is little difference in proliferation on poly-HEMA-coated plates.

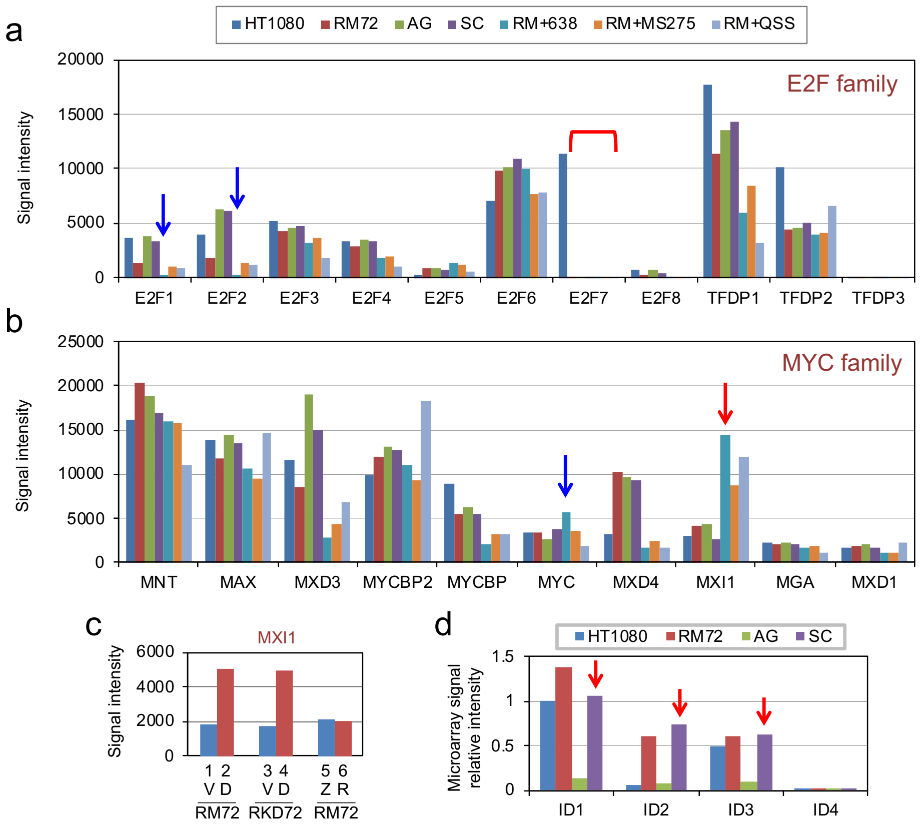

Supplementary Figure S7. Expression of *E2F*, *MYC*, and *ID* family genes. All data are based on transcriptome studies. (**a**) *E2F* family genes expressed in HT1080, RM72, AG, SC, and RM72 treated with DSK638, MS275, or QSS. Note the strong downregulation of *E2F1* and *E2F2* in RM72 cells in the presence of DSK638 (blue arrows) and the absence of *E2F7* signals in RM72 and its subpopulations, AG and SC (red bracket). (**b**) Expression of *MYC* family genes in the same set of cells as in panel a. Note the upregulation of *MYC* (blue arrow) and the prominent upregulation of *MXI1* (red arrow) in RM72 cells in the presence of DSK638. (**c**) Effects of DSK638 and RECK on *MXI1* expression. Expression of *MXI1* in RM72 cells (lanes 1, 2, 5, 6) and RKD72 cells (RECK-depleted RM72 cells; lanes 3, 4) treated with vehicle (V), DSK638 (D), control adenovirus (Z), or *RECK*-expressing adenovirus (R) is shown. Note that DSK638 upregulates *MXI1* even when RECK is depleted (lane 4) and that RECK overexpression fails to upregulate *MXI1*. (**d**) *ID* family genes expressed in HT1080, RM72, and its subpopulations, AG and SC. Note that *ID1*, *ID2*, and *ID3* are expressed in SC (red arrows) but that their expression is low AG (yellow green bars).

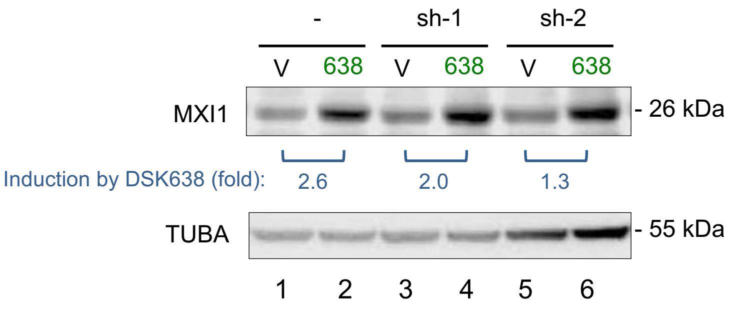

Supplementary Figure S8. MXI1 proteins in RM72 cells expressing small hairpin RNAs. RM72 cells (lanes 1, 2) and cells stably transfected with a vector expressing one of two small hairpin RNAs designed to knockdown MXI1 (sh-1, sh-2; lanes 3-6) incubated in medium containing vehicle (V) or DSK638 (638) were subjected to immunoblot assay using anti-MXI1 antibodies (top panel) and then α-tubulin (bottom panel). The relative intensity of each band was quantified using Image-J, and the data for MXI1 were divided by those for α-tubulin. The ratio between the calculated value for DSK638-treated cells and for vehicle-treated cells, representing the extent of induction by DSK638, are presented between the panels. Note that the induction of MXI1 by DSK638 is blunted in the cells expressing sh-2 (~1.3) compared to its induction in the parental cells (~2.6).

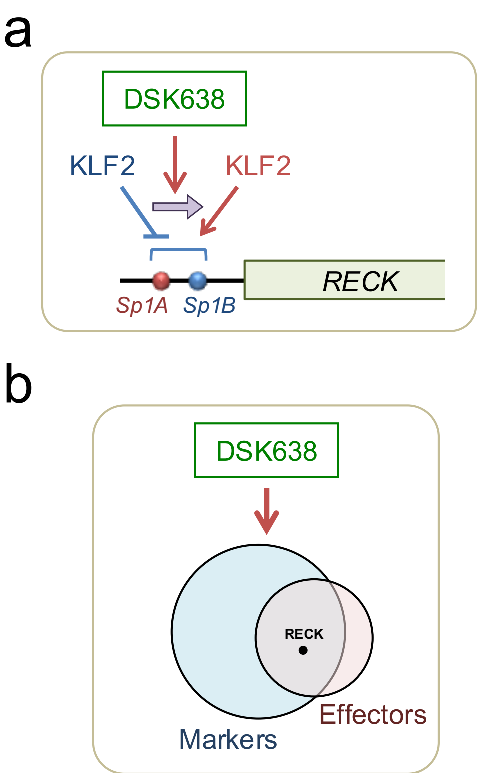

Supplementary Figure S9. (**a**) A model to explain how DSK638 upregulates RECK. (**b**) Schematic representation of the role of RECK as a marker (for cellular normalization) and an effector (in metastasis suppression) in our drug screening leading to the discovery of DSK638.

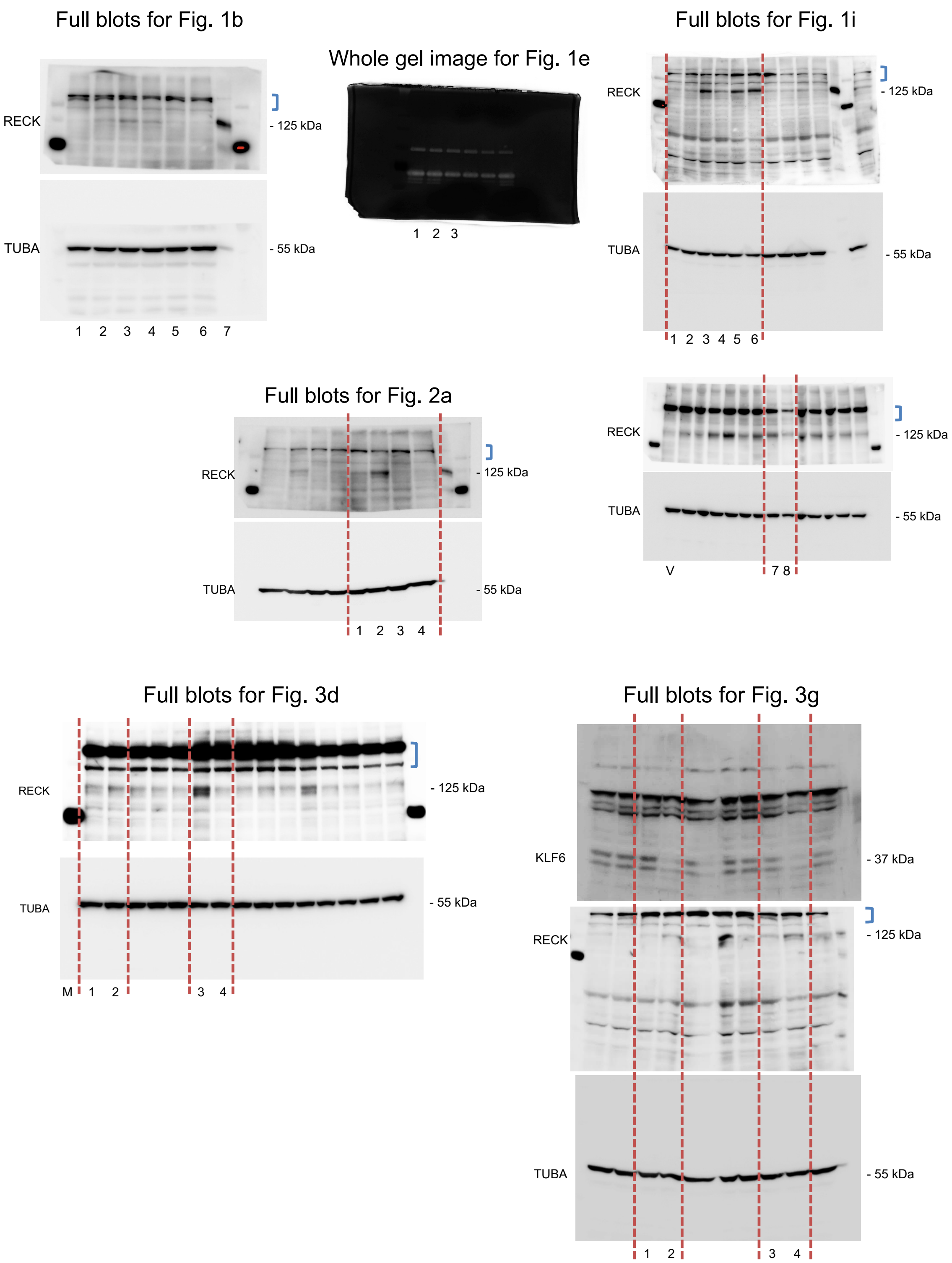

Supplementary Figure S10. Original blot and gel images (1/3). Blue brackets indicate background bands.

Full blots for Fig. 3i

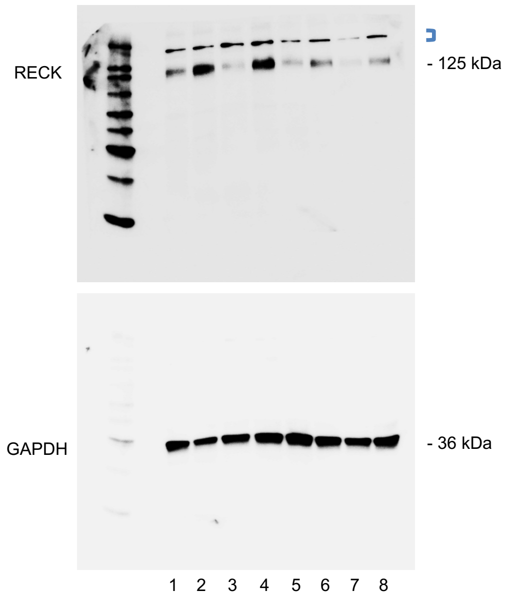

Full blots for Fig. 3k

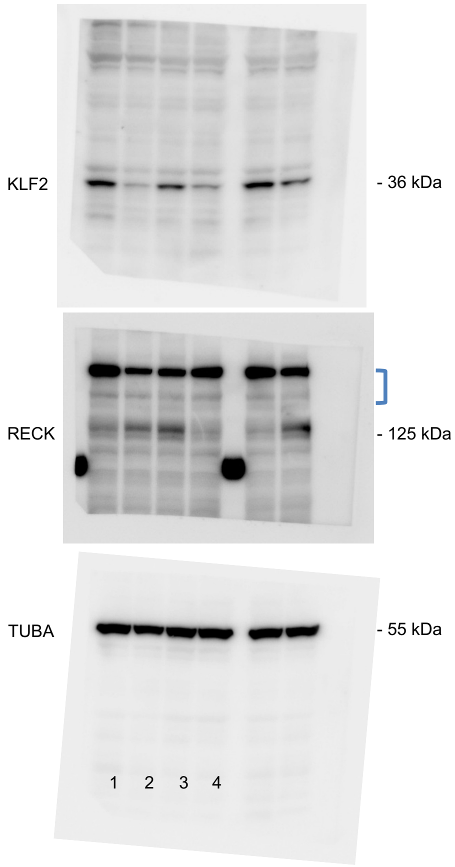

Full blots for Fig. 4f

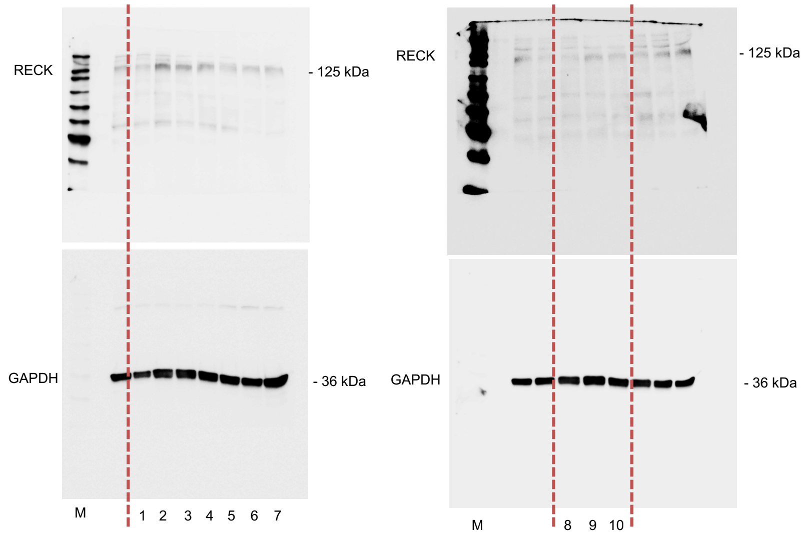

Supplementary Figure S10. Original blot and gel images (2/3).
Blue brackets indicate background bands

Full blots for Fig. 4I

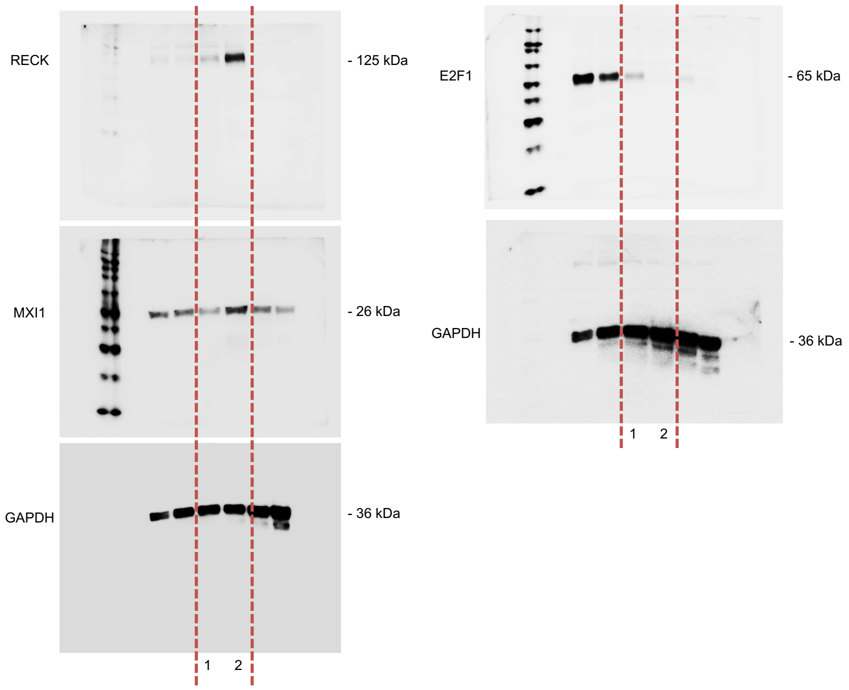

Full blots for Fig. S8

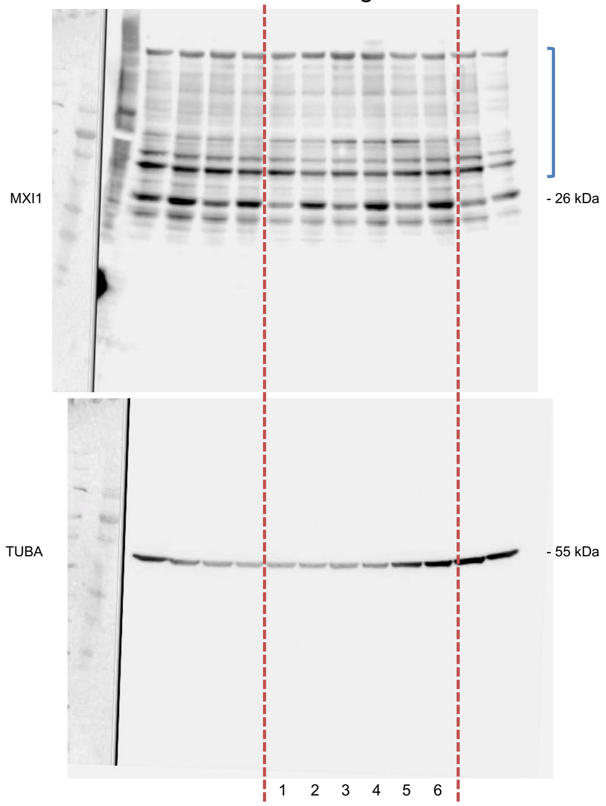

Supplementary Figure S10. Original blot images (3/3).

Blue bracket indicates background bands
