# Supplementary material for: Suppression of tumor metastasis by a RECK-activating small molecule
Source: Sci Rep. 2022 Feb 11;12:2319. doi: 10.1038/s41598-022-06288-3 (PMC8837781; doi:10.1038/s41598-022-06288-3)

Supplementary Table S1. Chemicals and antibodies

## A. Chemicals

| No | Name | Source | Cat. No. |
|---|---|---|---|
| 1 | DSK637 | Sumika Techno Service | |
| 2 | DSK638 | Sumika Techno Service | |
| 3 | N-(2-aminophenyl)benzamide [NAPB] | Santa Cruz | sc-354257 |
| 4 | N-phenyl-4-methylbenzamide [NPMB] | Alfa Aesar | H57070 |
| 5 | N-(2-Hydroxyphenyl)-4-methylbenzamide [NHPMB] | Sigma-Aldrich | R547107-1EA |
| 6 | N-(p-tolyl)-2-aminobenzamide [NTAB] | Santa Cruz | sc-274302 |
| 7 | CI-994 | Selleck | S2818 |
| 8 | MS275 | Selleck | S1053 |
| 9 | RGFP966 | Selleck | S7229 |
| 10 | Quisinostat (QSS) | Selleck | S1096 |

## B. Antibodies

| Antigen | Provider | Code No. | Host animal | Type | Band size (kDa) |
|---|---|---|---|---|---|
| Primary | | | | | |
| RECK | (original)* | 5B11D12 | mouse | monoclonal | 125 |
| KLF2 | Abnova | H00010365-A01 | mouse | polyclonal | 36 |
| KLF6 | Santa Cruz | sc-7158 | rabit | polyclonal | 37 |
| MXI1 | Santa Cruz | sc-130627 | mouse | monoclonal | 40** |
| E2F1 | CST | 3742 | rabbit | polyclonal | 65 |
| $\alpha$-tubulin | Calbiochem | CP06 | mouse | monoclonal | 55 |
| GAPDH | Santa Cruz | sc-20357 | goat | polyclonal | 36 |
| Secondary | | | | | |
| rabbit IgG | CST | 7074 | goat | HRP-linked | |
| mouse IgG | CST | 7076 | horse | HRP-linked | |

* see Takahashi et al. 1998

** fusion protein; native, ca. 26 kDa

Supplementary Table S2. Similarity to DSK638 in the spectrum of growth inhibition on a series of cancer-derived cell lines

| | Chemical | Target | Similartiy* (r) |
|---|---|---|---|
| 1 | PCI-24781 | HDACs (1, 2, 3, 6, 8) | 0.712 |
| 2 | Resminostat | HDACs (1, 3, 6) | 0.646 |
| 3 | SB939 | HDACs (1-5, 8, 10, 11) | 0.642 |
| 4 | Rocilinostat/ACY-1215 | HDACs (1-3, 6, 8) | 0.606 |
| 5 | LBH-589 | HDAC | 0.605 |
| 6 | Belinostat | HDAC | 0.586 |
| 7 | AZ-3146 | Mps1 (kinase) | 0.579 |
| 8 | Apicidin | HDAC | 0.579 |
| 9 | Entinostat (MS275) | HDACs (1, 3) | 0.579 |
| 10 | Scriptaid | HDACs (3, 4) | 0.578 |
| 11 | Givinostat | HDAC<br>IL-1$\beta$ production<br>TNF-$\alpha$ release | 0.561 |
| 12 | AG126 | TNF-$\alpha$ production | 0.535 |
| 13 | Dobutamine hydrochloride | $\beta$1-adrenergic receptors | 0.533 |
| 14 | Mesoprocol | IGF1R<br>Lipoxygenase | 0.53 |
| 15 | p, p'-DTT | $Na^+$ channel | 0.53 |
| 16 | JNJ-26481585 (QSS) | HDAC1 | 0.528 |
| 17 | CUDC-101 | EGFR<br>HER2<br>HDAC | 0.526 |
| 18 | Fenretinide | RAR$\beta$<br>RAR$\gamma$ | 0.516 |
| 19 | Dopamine hydrochloride | (Neurotransmitter) | 0.514 |
| 20 | Paclitaxel | Tubulin disassembly | 0.511 |

*Pearson correlation coefficient (r) between the distribution of growth inhibitory activity (GI50 values) of DSK638 on JFCR39 (a panel of 39 cancer-derived cell lines) and that of the indicated drug (see Akatsuka et al. 2016).

Supplementary Table S3. Cell inhibition, RECK induction, and HDAC inhibition by DSK638-related compounds

| No. | Code | Cell inhibition (IC50; μM) | | | | RECK induction (fold) | HDAC inhibition (IC50; μM) | |
|---|---|---|---|---|---|---|---|---|
| | | HT1080 | | RM72 | | | | |
| | | regular | polyHEMA | regular | polyHEMA | | HDAC1 | HDAC3 |
| 1 | DSK637 | >100 | >100 | >100 | >100 | 1.2 | >100 | >100 |
| 2 | DSK638 | 34 | 15 | 6.5 | 10 | 3.2 | 5 | 10 |
| 3 | NAPB | >100 | 98 | 44 | >100 | 1.3 | 8 | 5 |
| 4 | NPMB | >100 | >100 | >100 | >100 | 0.4 | >100 | > 100 |
| 5 | NHPMB | >100 | >100 | 78 | 98 | 0.3 | 3 | 3 |
| 6 | NTAB | >100 | >100 | >100 | >100 | 0.6 | > 100 | > 100 |
| 7 | CI-994 | 29 | 58 | 8.1 | 78 | 2.6 | 0.9 | 1.2 |
| 8 | MS275 | 3.8 | 29 | 2.8 | 0.52 | 3.7 | 0.51 | 1.7 |
| 9 | RGFP966 | 94 | >100 | 39 | >100 | 0.6 | >15 | 0.08 |
| 10 | QSS | 0.9 | 0.49 | 0.38 | 0.084 | 3.0 | 0.0001 | 0.0005 |

Supplementary Table S4. GSEA: SC vs. AG (higher in SC; 6230 genes)

| | NAME | SIZE | ES | NES | NOM p-val | FDR q-val | FWER p-val | RANK AT MAX | LEADING EDGE |
|---|---|---|---|---|---|---|---|---|---|
| 1 | REGULATION_OF_TRANSCRIPTION_FACTOR_ACTIVITY | 15 | 0.79 | 1.87 | 0.004 | 0.139 | 0.103 | 20 | tags=20%, list=0%, signal=20% |
| 2 | REGULATION_OF_DNA_BINDING | 18 | 0.75 | 1.86 | 0.005 | 0.086 | 0.125 | 20 | tags=17%, list=0%, signal=17% |
| 3 | NEGATIVE_REGULATION_OF_METABOLIC_PROCESS | 106 | 0.54 | 1.84 | 0.000 | 0.075 | 0.162 | 555 | tags=21%, list=9%, signal=22% |
| 4 | NEGATIVE_REGULATION_OF_CELLULAR_METABOLIC_PROCESS | 104 | 0.53 | 1.83 | 0.002 | 0.068 | 0.194 | 555 | tags=20%, list=9%, signal=22% |
| 5 | NEGATIVE_REGULATION_OF_TRANSCRIPTION | 76 | 0.54 | 1.80 | 0.000 | 0.082 | 0.273 | 555 | tags=20%, list=9%, signal=21% |
| 6 | REGULATION_OF_BINDING | 23 | 0.69 | 1.79 | 0.004 | 0.072 | 0.284 | 20 | tags=13%, list=0%, signal=13% |
| 7 | NEGATIVE_REGULATION_OF_NUCLEOBASENUCLEOSIDENUCLEOTIDE_AND_NUCLEIC_ACID_METABOLIC_PROCESS | 81 | 0.54 | 1.78 | 0.002 | 0.069 | 0.301 | 555 | tags=20%, list=9%, signal=21% |
| 8 | CELLULAR_COMPONENT_DISASSEMBLY | 19 | 0.68 | 1.68 | 0.015 | 0.181 | 0.676 | 559 | tags=21%, list=9%, signal=23% |
| 9 | TISSUE_DEVELOPMENT | 38 | 0.58 | 1.64 | 0.013 | 0.234 | 0.808 | 575 | tags=32%, list=9%, signal=35% |
| 10 | NEURON_DEVELOPMENT | 22 | 0.62 | 1.59 | 0.023 | 0.342 | 0.935 | 712 | tags=41%, list=11%, signal=46% |
| 11 | NEGATIVE_REGULATION_OF_CELLULAR_PROCESS | 257 | 0.40 | 1.53 | 0.002 | 0.497 | 0.986 | 555 | tags=14%, list=9%, signal=15% |
| 12 | NEGATIVE_REGULATION_OF_BIOLOGICAL_PROCESS | 268 | 0.39 | 1.50 | 0.000 | 0.545 | 0.994 | 957 | tags=22%, list=15%, signal=24% |
| 13 | REGULATION_OF_MOLECULAR_FUNCTION | 103 | 0.43 | 1.45 | 0.019 | 0.608 | 0.999 | 976 | tags=22%, list=16%, signal=26% |
| 14 | REGULATION_OF_METABOLIC_PROCESS | 288 | 0.35 | 1.38 | 0.003 | 0.706 | 1.000 | 918 | tags=19%, list=15%, signal=21% |
| 15 | REGULATION_OF_GENE_EXPRESSION | 241 | 0.36 | 1.37 | 0.007 | 0.675 | 1.000 | 847 | tags=17%, list=14%, signal=19% |
| 16 | REGULATION_OF_TRANSCRIPTION | 210 | 0.36 | 1.36 | 0.018 | 0.608 | 1.000 | 847 | tags=17%, list=14%, signal=19% |
| 17 | CARBOHYDRATE_METABOLIC_PROCESS | 71 | 0.43 | 1.36 | 0.044 | 0.593 | 1.000 | 1284 | tags=38%, list=21%, signal=47% |
| 18 | REGULATION_OF_CELLULAR_METABOLIC_PROCESS | 282 | 0.35 | 1.34 | 0.007 | 0.564 | 1.000 | 918 | tags=19%, list=15%, signal=21% |
| 19 | MULTICELLULAR_ORGANISMAL_DEVELOPMENT | 330 | 0.33 | 1.32 | 0.021 | 0.585 | 1.000 | 656 | tags=20%, list=11%, signal=21% |
| 20 | REGULATION_OF_NUCLEOBASENUCLEOSIDENUCLEOTIDE_AND_NUCLEIC_ACID_METABOLIC_PROCESS | 227 | 0.35 | 1.30 | 0.035 | 0.566 | 1.000 | 570 | tags=12%, list=9%, signal=13% |
| 21 | INTRACELLULAR_SIGNALING_CASCADE | 206 | 0.33 | 1.25 | 0.044 | 0.555 | 1.000 | 976 | tags=25%, list=16%, signal=28% |
| 22 | TRANSCRIPTION | 285 | 0.31 | 1.22 | 0.041 | 0.539 | 1.000 | 847 | tags=15%, list=14%, signal=17% |

Supplementary Table S5. GSEA details: NEGATIVE_REGULATION_OF_METABOLIC_PROCESS

Leading-edge subset

| | GENE SYMBOL | RANK IN GENE LIST | RANK METRIC SCORE | RUNNING ES |
|---|---|---|---|---|
| 1 | ID2 | 13 | 3.011 | 0.0745 |
| 2 | ID1 | 15 | 2.929 | 0.1488 |
| 3 | TNP1 | 16 | 2.919 | 0.223 |
| 4 | ID3 | 20 | 2.624 | 0.2892 |
| 5 | FOSB | 52 | 1.792 | 0.3297 |
| 6 | NRG1 | 76 | 1.32 | 0.3596 |
| 7 | IL6 | 83 | 1.265 | 0.3908 |
| 8 | INHBB | 108 | 1.057 | 0.4137 |
| 9 | CDA | 129 | 0.958 | 0.4348 |
| 10 | SNAI2 | 173 | 0.834 | 0.449 |
| 11 | GHRL | 198 | 0.771 | 0.4647 |
| 12 | CALCA | 251 | 0.678 | 0.4734 |
| 13 | VDR | 267 | 0.661 | 0.4878 |
| 14 | BCOR | 371 | 0.512 | 0.484 |
| 15 | CDKN2A | 381 | 0.504 | 0.4953 |
| 16 | GLA | 449 | 0.45 | 0.4959 |
| 17 | ZNF157 | 464 | 0.439 | 0.5047 |
| 18 | GCLC | 519 | 0.409 | 0.5063 |
| 19 | PAWR | 532 | 0.406 | 0.5147 |
| 20 | NRIP1 | 553 | 0.398 | 0.5215 |
| 21 | FST | 555 | 0.397 | 0.5315 |

GSEA results summary

| GeneSet | NEGATIVE_REGULATION_OF_METABOLIC_PROCESS |
|---|---|
| ES | 0.537 |
| NES | 1.840 |
| Nominal p-value | 0.000 |
| FDR q-value | 0.075 |
| FWER p-Value | 0.162 |

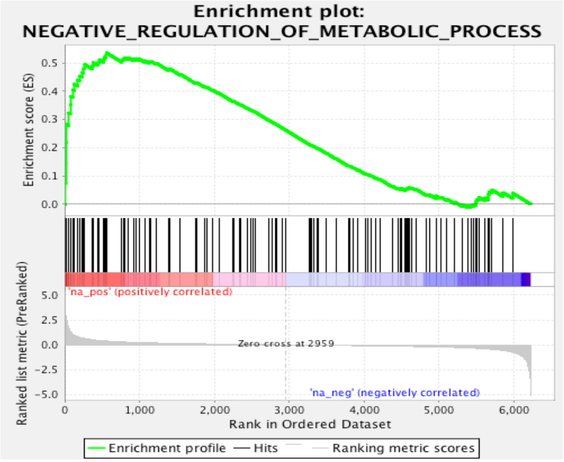


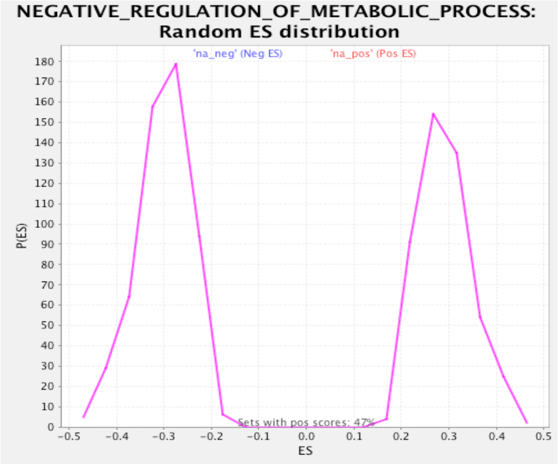

Supplementary Table S6. GSEA: Effects of DSK638 on RM72 cells (upregulated; 6201 genes)

| | NAME | SIZE | ES | NES | NOM p-val | FDR q-val | FWER p-val | RANK AT MAX | LEADING EDGE |
|---|---|---|---|---|---|---|---|---|---|
| 1 | BEHAVIOR | 39 | 0.76 | 1.94 | 0.000 | 0.001 | 0.001 | 494 | tags=36%, list=8%, signal=39% |
| 2 | EXTRACELLULAR_STRUCTURE_ORGANIZATION_AND_BIOGENESIS | 16 | 0.86 | 1.92 | 0.000 | 0.001 | 0.002 | 494 | tags=63%, list=8%, signal=68% |
| 3 | LOCOMOTORY_BEHAVIOR | 25 | 0.79 | 1.91 | 0.000 | 0.001 | 0.002 | 454 | tags=44%, list=7%, signal=47% |
| 4 | SYNAPTIC_TRANSMISSION | 53 | 0.70 | 1.88 | 0.000 | 0.001 | 0.005 | 498 | tags=28%, list=8%, signal=31% |
| 5 | TRANSMISSION_OF_NERVE_IMPULSE | 60 | 0.68 | 1.80 | 0.000 | 0.006 | 0.032 | 591 | tags=28%, list=10%, signal=31% |
| 6 | FEMALE_PREGNANCY | 16 | 0.80 | 1.76 | 0.001 | 0.013 | 0.084 | 494 | tags=44%, list=8%, signal=47% |
| 7 | G_PROTEIN_COUPLED_RECEPTOR_PROTEIN_SIGNALING_PATHWAY | 73 | 0.62 | 1.70 | 0.001 | 0.033 | 0.231 | 611 | tags=29%, list=10%, signal=32% |
| 8 | CELL_CELL_SIGNALING | 131 | 0.60 | 1.69 | 0.000 | 0.032 | 0.248 | 1083 | tags=33%, list=17%, signal=39% |

Supplementary Table S7. GSEA details: EXTRACELLULAR STRUCTURE ORGANIZATION AND BIOGENESIS

Leading-edge subset

| | GENE SYMBOL | RANK IN GENE LIST | RANK METRIC SCORE | RUNNING ES |
|---|---|---|---|---|
| 1 | KLK8 | 4 | 7.316 | 0.1616 |
| 2 | PCDHB10 | 28 | 6.018 | 0.2914 |
| 3 | PCDHB5 | 33 | 5.821 | 0.4199 |
| 4 | PCDHB9 | 62 | 5.005 | 0.5264 |
| 5 | PCDHB6 | 93 | 4.522 | 0.6218 |
| 6 | PCDHB2 | 175 | 3.609 | 0.6888 |
| 7 | PCDHB11 | 214 | 3.315 | 0.7562 |
| 8 | PCDHB14 | 379 | 2.518 | 0.7855 |
| 9 | PCDHB4 | 417 | 2.321 | 0.831 |
| 10 | GHRL | 494 | 2.057 | 0.8644 |

GSEA results summary

| GeneSet | EXTRACELLULAR STRUCTURE ORGANIZATION AND BIOGENESIS |
|---|---|
| ES | 0.864 |
| NES | 1.920 |
| Nominal p-value | 0.000 |
| FDR q-value | 0.001 |
| FWER p-Value | 0.002 |

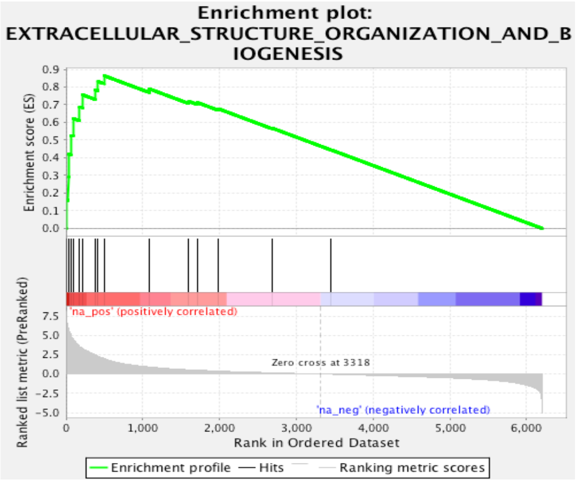


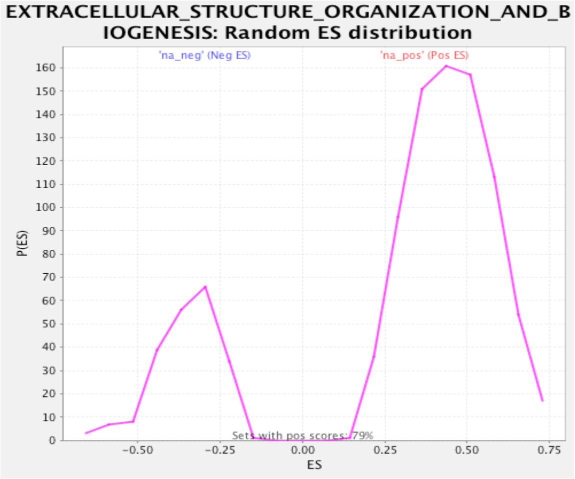

Supplementary Table S8. GSEA: Effects of DSK638 on RM72 cells (downregulated; 2088 genes)

| | NAME | SIZE | ES | NES | NOM p-val | FDR q-val | FWER p-val | RANK AT MAX | LEADING EDGE |
|---|---|---|---|---|---|---|---|---|---|
| 1 | DNA_METABOLIC_PROCESS | 40 | −0.63 | −3.83 | 0.000 | 0.000 | 0.000 | 403 | tags=73%, list=19%, signal=88% |
| 2 | MITOSIS | 29 | −0.70 | −3.75 | 0.000 | 0.000 | 0.000 | 313 | tags=76%, list=15%, signal=88% |
| 3 | M_PHASE_OF_MITOTIC_CELL_CYCLE | 31 | −0.67 | −3.66 | 0.000 | 0.000 | 0.000 | 313 | tags=71%, list=15%, signal=82% |
| 4 | RESPONSE_TO_DNA_DAMAGE_STIMULUS | 25 | −0.72 | −3.55 | 0.000 | 0.000 | 0.000 | 403 | tags=84%, list=19%, signal=103% |
| 5 | DNA_REPAIR | 21 | −0.74 | −3.52 | 0.000 | 0.000 | 0.000 | 397 | tags=86%, list=19%, signal=105% |
| 6 | MITOTIC_CELL_CYCLE | 48 | −0.53 | −3.45 | 0.000 | 0.000 | 0.000 | 313 | tags=58%, list=15%, signal=67% |
| 7 | RESPONSE_TO_ENDOGENOUS_STIMULUS | 27 | −0.64 | −3.36 | 0.000 | 0.000 | 0.000 | 403 | tags=78%, list=19%, signal=95% |
| 8 | CELL_CYCLE_PROCESS | 58 | −0.46 | −3.16 | 0.000 | 0.000 | 0.000 | 317 | tags=57%, list=15%, signal=65% |
| 9 | M_PHASE | 38 | −0.52 | −3.09 | 0.000 | 0.000 | 0.000 | 313 | tags=68%, list=15%, signal=79% |
| 10 | CELL_CYCLE_GO_0007049 | 77 | −0.42 | −3.03 | 0.000 | 0.000 | 0.000 | 324 | tags=52%, list=16%, signal=59% |
| 11 | CELL_CYCLE_PHASE | 52 | −0.45 | −2.93 | 0.000 | 0.000 | 0.000 | 313 | tags=56%, list=15%, signal=64% |
| 12 | CHROMOSOME_ORGANIZATION_AND_BIOG | 20 | −0.60 | −2.86 | 0.000 | 0.000 | 0.000 | 481 | tags=75%, list=23%, signal=97% |
| 13 | DNA_REPLICATION | 16 | −0.63 | −2.79 | 0.000 | 0.000 | 0.000 | 403 | tags=75%, list=19%, signal=92% |
| 14 | CHROMOSOME_SEGREGATION | 15 | −0.66 | −2.64 | 0.000 | 0.000 | 0.000 | 285 | tags=67%, list=14%, signal=77% |
| 15 | NEGATIVE_REGULATION_OF_NUCLEOBASE | 21 | −0.52 | −2.43 | 0.000 | 0.000 | 0.004 | 430 | tags=62%, list=21%, signal=77% |
| 16 | NEGATIVE_REGULATION_OF_TRANSCRIPTI | 20 | −0.50 | −2.39 | 0.000 | 0.001 | 0.005 | 430 | tags=60%, list=21%, signal=75% |
| 17 | NUCLEOBASENUCLEOSIDENUCLEOTIDE_A | 125 | −0.31 | −2.38 | 0.000 | 0.001 | 0.005 | 407 | tags=42%, list=19%, signal=49% |

Supplementary Table S9. GSEA details: MITOSIS (downregulated by DSK638)

Leading–edge subset

| | GENE SYMBOL* | RANK IN GENE LIST | RANK METRIC SCORE | RUNNING ES |
|---|---|---|---|---|
| 14 | CDC25B | 1545 | −2.369 | -0.520 |
| 15 | NUMA1 | 1561 | −2.386 | -0.512 |
| 16 | CDK6 | 1584 | −2.419 | -0.508 |
| 17 | DLGAP5 | 1606 | −2.448 | -0.503 |
| 18 | SMC4 | 1624 | −2.48 | -0.495 |
| 19 | CDK10 | 1676 | −2.601 | −0.504 |
| 20 | CDKN2C | 1722 | −2.748 | −0.509 |
| 21 | TPX2 | 1776 | −2.908 | −0.517 |
| 22 | CDC25C | 1797 | −2.969 | −0.508 |
| 23 | NUSAP1 | 1804 | −2.984 | −0.492 |
| 24 | KIF2C | 1813 | −3.027 | −0.477 |
| 25 | PRC1 | 1835 | −3.142 | −0.467 |
| 26 | CENPF | 1843 | −3.183 | −0.451 |
| 27 | KIF22 | 1878 | −3.364 | −0.447 |
| 28 | UBE2C | 1879 | −3.364 | −0.425 |
| 29 | ANLN | 1886 | −3.398 | −0.407 |
| 30 | CENPE | 1916 | −3.557 | −0.399 |
| 31 | CIT | 1918 | −3.563 | −0.377 |
| 32 | KIF23 | 1922 | −3.579 | −0.356 |
| 33 | KIF11 | 1939 | −3.688 | −0.341 |
| 34 | BUB1B | 1942 | −3.724 | −0.318 |
| 35 | E2F1 | 1959 | −3.839 | −0.302 |
| 36 | POLE | 1964 | −3.884 | −0.2796 |
| 37 | ESPL1 | 1968 | −3.902 | −0.2566 |
| 38 | MAD2L1 | 1972 | −3.962 | −0.2332 |
| 39 | AURKA | 1983 | −4.144 | −0.2121 |
| 40 | BUB1 | 1986 | −4.161 | −0.1869 |
| 41 | ZWINT | 1987 | −4.163 | −0.1608 |
| 42 | FBXO5 | 2002 | −4.287 | −0.1407 |
| 43 | PLK1 | 2011 | −4.403 | −0.117 |
| 44 | PKMYT1 | 2012 | −4.408 | −0.0894 |
| 45 | KIF15 | 2021 | −4.558 | −0.0647 |
| 46 | NDC80 | 2024 | −4.591 | −0.0368 |
| 47 | CDCA5 | 2050 | −5.305 | −0.0158 |
| 48 | TTK | 2051 | −5.326 | 0.0176 |

*Genes common in Tables 9 and 11 are highlighted in red.

GSEA results summary

| GeneSet | MITOSIS |
|---|---|
| ES | -0.535 |
| NES | -3.450 |
| Nominal p-value | 0.000 |
| FDR q-value | 0.000 |
| FWER p-Value | 0.000 |

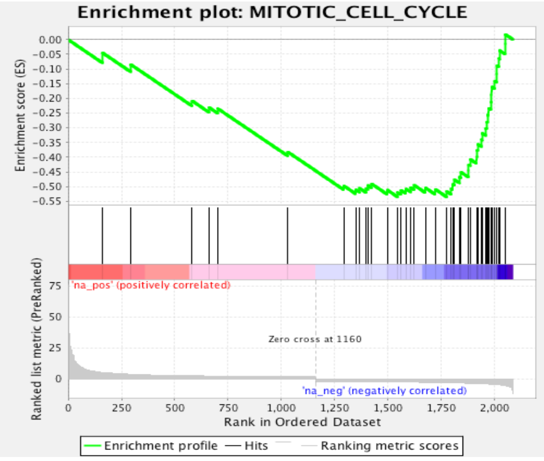


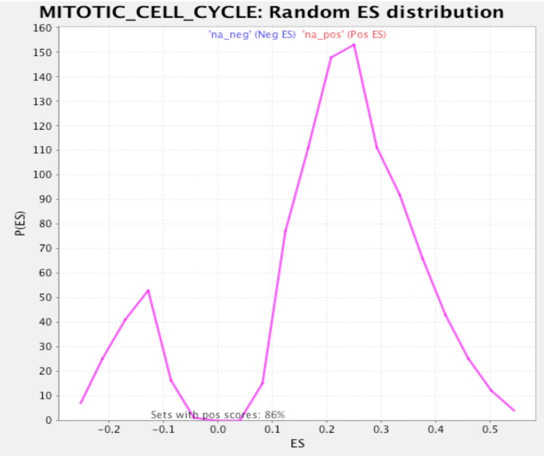

Supplementary Table S10. GSEA: RM72 vs. HT1080 (lower in RM72; 6484 genes)

| | NAME | SIZE | ES | NES | NOM p-val | FDR q-val | FWER p-val | RANK AT MAX | LEADING EDGE |
|---|---|---|---|---|---|---|---|---|---|
| 1 | MITOTIC_CELL_CYCLE | 70 | -0.71 | -2.32 | 0.000 | 0.000 | 0.000 | 760 | tags=46%, list=12%, signal=51% |
| 2 | CELL_CYCLE_PROCESS | 86 | -0.67 | -2.22 | 0.000 | 0.000 | 0.000 | 408 | tags=38%, list=6%, signal=40% |
| 3 | M_PHASE_OF_MITOTIC_CELL_CYCLE | 38 | -0.76 | -2.20 | 0.000 | 0.000 | 0.000 | 364 | tags=47%, list=6%, signal=50% |
| 4 | CELL_CYCLE_PHASE | 77 | -0.66 | -2.17 | 0.000 | 0.000 | 0.000 | 408 | tags=39%, list=6%, signal=41% |
| 5 | MITOSIS | 38 | -0.76 | -2.16 | 0.000 | 0.000 | 0.000 | 364 | tags=47%, list=6%, signal=50% |
| 6 | REGULATION_OF_MITOSIS | 19 | -0.84 | -2.13 | 0.000 | 0.000 | 0.000 | 364 | tags=58%, list=6%, signal=61% |
| 7 | M_PHASE | 51 | -0.68 | -2.11 | 0.000 | 0.000 | 0.000 | 408 | tags=45%, list=6%, signal=48% |
| 8 | CELL_CYCLE_GO_0007049 | 136 | -0.58 | -2.08 | 0.000 | 0.000 | 0.000 | 453 | tags=30%, list=7%, signal=32% |
| 9 | REGULATION_OF_CELL_CYCLE | 76 | -0.61 | -2.04 | 0.000 | 0.000 | 0.001 | 799 | tags=38%, list=12%, signal=43% |

Supplementary Table S11. GSEA details: MITOTIC_CELL_CYCLE (lower in RM72)

Leading-edge subset

| | GENE SYMBOL* | RANK IN GENE LIST | RANK METRIC SCORE | RUNNING ES |
|---|---|---|---|---|
| 32 | BTG3 | 5725 | −1.149 | −0.700 |
| 31 | APBB1 | 5741 | −1.168 | −0.692 |
| 30 | GFI1 | 5760 | −1.193 | −0.683 |
| 29 | ZW10 | 5808 | −1.269 | −0.678 |
| 28 | E2F1 | 5945 | −1.533 | −0.685 |
| 27 | KPNA2 | 6078 | −1.871 | −0.688 |
| 26 | BUB1 | 6121 | −2.032 | −0.675 |
| 25 | KNTC1 | 6128 | −2.063 | −0.657 |
| 24 | SKP2 | 6158 | −2.168 | −0.641 |
| 23 | NEK2 | 6167 | −2.192 | −0.621 |
| 22 | CDC7 | 6205 | −2.413 | −0.604 |
| 21 | ZWINT | 6246 | −2.605 | −0.585 |
| 20 | TPX2 | 6252 | −2.633 | −0.561 |
| 19 | INHBA | 6264 | −2.707 | −0.537 |
| 18 | MAD2L1 | 6270 | −2.745 | −0.512 |
| 17 | CDC25C | 6278 | −2.775 | −0.487 |
| 16 | KIF23 | 6293 | −2.886 | −0.462 |
| 15 | ESPL1 | 6294 | −2.887 | −0.434 |
| 14 | KIF15 | 6295 | −2.892 | −0.407 |
| 13 | CDCA5 | 6305 | −2.959 | −0.380 |
| 12 | TTK | 6306 | −2.960 | −0.352 |
| 11 | CENPF | 6315 | −3.026 | −0.325 |
| 10 | BUB1B | 6318 | −3.051 | −0.296 |
| 9 | CDKN3 | 6324 | −3.082 | −0.268 |
| 8 | EGF | 6333 | −3.113 | −0.239 |
| 7 | ANLN | 6342 | −3.230 | −0.210 |
| 6 | CENPE | 6366 | −3.396 | −0.181 |
| 5 | KIF2C | 6370 | −3.439 | −0.149 |
| 4 | CCNA2 | 6394 | −3.685 | −0.118 |
| 3 | PLK1 | 6397 | −3.686 | −0.083 |
| 2 | PRC1 | 6410 | −3.850 | −0.049 |
| 1 | CDKN1C | 6469 | −6.314 | 0.002 |

*Genes common in Tables 9 and 11 are highlighted in red.

GSEA results summary

| GeneSet | MITOTIC CELL CYCLE |
|---|---|
| ES | -0.711 |
| NES | -2.319 |
| Nominal p-value | 0.000 |
| FDR q-value | 0.000 |
| FWER p-Value | 0.000 |

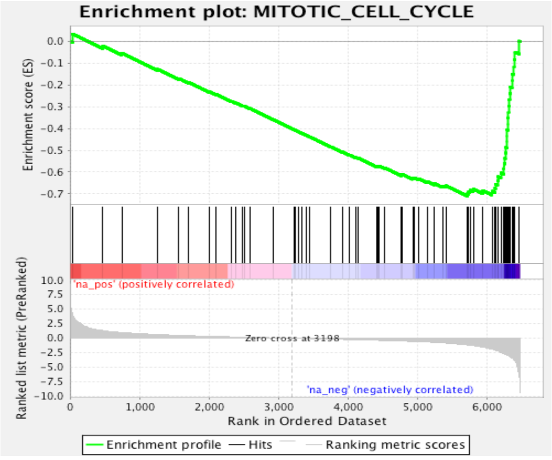


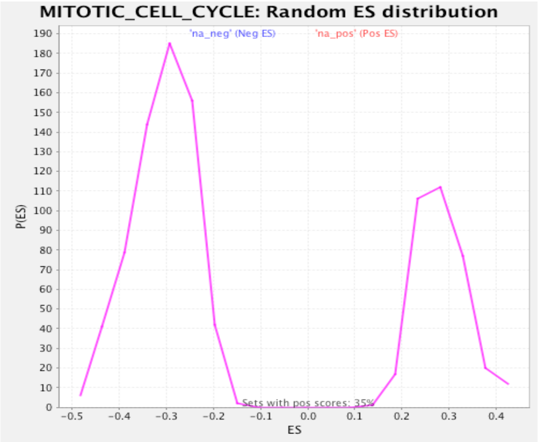

Supplement: Supplementary file 2 — Supplementary Information 2. [file 41598_2022_6288_MOESM2_ESM.pdf]
